# Environmental Drivers of Nontuberculous Mycobacteria in Tropical Freshwater Systems

**DOI:** 10.1111/1462-2920.70465

**Published:** 2026-11-04

**Authors:** Marine Combe, Amar Bouam, Sylvestre Dizoe, Bernard Davoust, Emilien Drancourt, Dylan Messeca, Alice Valentini, Romain Blaizot, Rodolphe Elie Gozlan

**Affiliations:** ^1^ ISEM, Université de Montpellier, CNRS, IRD, EPHE Montpellier France; ^2^ Aix‐Marseille Univ., IRD, MEPHI, IHU Méditerranée Infection Marseille France; ^3^ Programme National de Lutte Contre l'Ulcère de Buruli (PNLUB), Kongouanou Care Center Yamoussoukro Côte d'Ivoire; ^4^ Aix‐Marseille Univ., RITMES, AP‐HM, SSA, IHU Méditerranée Infection Marseille France; ^5^ SPYGEN Le Bourget‐du‐Lac France; ^6^ Service de Dermatologie Centre Hospitalier Andrée Rosemon Cayenne Guyane Française France

**Keywords:** aquatic, bacterial, biogeography, ecology, eDNA

## Abstract

Whilst microbial diversity still remains largely unexplored across environments and scales, microbial biogeography is shaped by edaphic and ecological drivers, and shifts in microbial community composition are frequently associated with host community structure and health. Nontuberculous mycobacteria represent a striking example of environmental microorganisms with opportunistic pathogenic potential. Unfortunately, understanding of their diversity and distribution in tropical freshwater environments remains limited, while such knowledge is crucial for predicting disease emergence and transmission. Here we aimed at (i) identifying microhabitat abiotic and biotic drivers influencing their diversity and distribution in French Guiana, (ii) comparing their diversity and distribution between French Guiana and Côte d'Ivoire, and (iii) examining co‐occurrences between mycobacterial communities in different environmental contexts. By deploying an eDNA‐based metabarcoding approach from tropical freshwater ecosystems, we suggest that their diversity and distribution are mainly driven by habitat type (urban vs. rural), sample type (water vs. sediment), and local abiotic factors such as phosphorus levels in sediments (with species‐specific effects). Moreover, we found a stratified distribution of mycobacteria depending on sediment texture (fine silts vs. coarse sand) and depth. Overall, we suggest that their ecological interactions may occur at the microhabitat level rather than following clear biogeographic patterns.

## Graphical abstract

In tropical freshwater ecosystems, nontuberculous mycobacterial diversity and distribution is mainly driven by habitat and sample type, and local environmental factors such as sediment texture and depth. These findings highlight the importance of microhabitat conditions over broad biogeographic patterns in shaping mycobacterial communities.

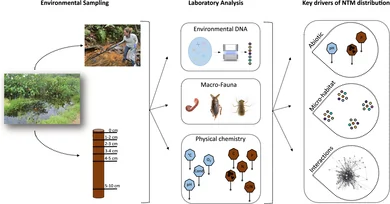

## 1 Introduction

Anthropogenic pressures, including climate change, deforestation, intensive agriculture, and urban expansion are key drivers of biodiversity changes and shifts in microbial community compositions, ultimately influencing the emergence of infectious diseases (Daszak et al. 2000; Morris et al. 2014, 2016; Wu et al. 2016; Cable et al. 2017; Stat et al. 2017; Reverter et al. 2020). In ecological theory, biodiversity is recognised as fundamental to ecosystem resilience (Stat et al. 2017; Jagadesh, Combe, Couppié, Nacher, and Gozlan 2019; Jagadesh, Combe, Couppié, Le Turnier, et al. 2019) and has been hypothesised to mitigate infectious disease risks through the ‘dilution effect’ although this remains debated (Keesing et al. 2006; Keesing and Ostfeld 2021).

Tropical freshwater ecosystems illustrate these dynamics, where for example deforestation‐driven aquatic food web alterations reduce top fish predators, favouring the proliferation of low‐trophic taxa that harbour *Mycobacterium ulcerans* ( *M. ulcerans* ), the causative agent of Buruli ulcer, thereby increasing human exposure risk to the pathogen (Morris et al. 2016). This illustrates the links between biodiversity loss, ecosystem health, and human health. Also, these environmental shifts align with the pathobiome concept in human and animal health, where microbiome disturbances promote pathogen proliferation (Bass et al. 2019; Pitlik and Koren 2017). Pathogenic transitions from diverse, stable microbiomes to dysbiotic states occur under multiple abiotic and biotic stressors, including temperature, host immune responses, and microbiota composition (Lai et al. 2015). As such, unravelling microbial diversity and interactions is not only fundamental to microbial biogeography and community assembly but also to understanding the relationships between biodiversity, ecosystem stability, and health within the One Health framework (Combe and Gozlan 2024).

Microbial diversity remains largely unexplored across environments and scales (Curtis et al. 2002). The classical principle proposed by Baas Becking (1934), ‘Everything is everywhere, but, the environment selects,’ suggests that microorganisms are globally distributed but often remain under a dormant state, until specific abiotic or biotic conditions trigger their proliferation (Wit and Bouvier 2006). At local scales, dormancy may imply that many microbial taxa exist below culture‐based detection, reinforcing the significant development of molecular approaches to characterise environmental microbial diversity. For instance, advances in environmental DNA (eDNA)‐based metabarcoding have significantly enhanced biosurveillance and biodiversity assessments across multiple habitats, including air, sediments, soil, and aquatic systems (Valentini et al. 2016; Drummond et al. 2015; Olds et al. 2016; Clare et al. 2021; Nuñez et al. 2021).

Microbial biogeography is shaped by edaphic and ecological drivers. For instance, soil bacterial diversity is primarily structured by pH, with higher diversity in neutral compared to acidic soils (Fierer and Jackson 2006). Similarly, shifts in microbial community composition are frequently associated with host community structure (Bass et al. 2019), which may influence pathogen dispersal; for example, the transmission of *M. ulcerans* is likely mediated through food web interactions (Combe et al. 2017; Hammoudi et al. 2020). Understanding the distribution of potential microbial pathogens depending on host (such as macrofauna) community structure is therefore essential for assessing biodiversity‐health relationships and predicting disease emergence risks.

Nontuberculous mycobacteria (NTM) represent an ideal example of environmental microorganisms with opportunistic pathogenic potential. These bacteria are widely distributed across ecosystems (Nichols et al. 2004; Combe et al. 2023) and act as opportunistic pathogens affecting human and animal activities, including natural waters (fresh, brackish, and marine), drinking water systems, soils (Falkinham 2002, 2009), aquaria, aquaculture reservoirs (Yanong et al. 2010), and wastewater (Amha et al. 2017; Combe unpublished data). Their associated diseases pose significant health risks and economic burdens (Douine et al. 2017), particularly in aquaculture and fish farming in developing countries (Phung et al. 2013).

Among NTM species infecting humans, the *Mycobacterium avium* complex (MAC) is most frequently implicated, causing lymphadenitis in children, respiratory infections in the elderly, and disseminated disease in immunocompromised individuals (Nichols et al. 2004). Other species, such as *Mycobacterium fortuitum* , *Mycobacterium abscessus* , *Mycobacterium chelonae* , and *Mycobacterium smegmatis* , are associated with skin infections (De Groote and Johnson 2004), while *Mycobacterium haemophilum* induces cutaneous and systemic infections, mainly in immunocompromised patients (Straus et al. 1994). Mainly in low‐ and middle‐income countries, mycolactone‐producing‐mycobacteria (MPM), including *M. ulcerans* , *Mycobacterium shinshuense* , *Mycobacterium liflandii* , and *Mycobacterium pseudoshottsii* , are responsible for Buruli ulcer, a debilitating skin disease (Douine et al. 2017; Combe et al. 2017, 2023). Notably, *M. gilvum* has been identified from cutaneous ulcers clinically mimicking those of Buruli ulcers in patients in French Guiana, sometimes as co‐infections with unidentified mycobacterial species (Combe et al. 2020).

Environmental factors are known to shape NTM proliferation in aquatic habitats rich in organic matter or animal excreta, as well as in disturbed or polluted swamps characterised by high temperatures, low pH, low dissolved oxygen, and elevated zinc levels (Oloya et al. 2008; Jagadesh, Combe, Couppié, Nacher, and Gozlan 2019; Jagadesh, Combe, Couppié, Le Turnier, et al. 2019). Furthermore, NTM species exhibit distinct environmental preferences (e.g., brackish vs. freshwater systems) and metabolic strategies (e.g., slow vs rapid growth) (Cangelosi et al. 2004), also suggesting that local selective pressures may constrain their geographic distribution. However, despite their health and economic impacts, empirical data on NTM diversity and distribution in freshwater tropical environments still remain limited. Understanding the role of abiotic (physico‐chemical parameters, granulometry) and biotic (macro‐fauna) factors shaping their biogeography is crucial for predicting disease emergence and transmission. This study aims to: (1) identify microhabitat abiotic drivers (pH, oxygen, conductivity, temperature, phosphorus, nitrogen, carbon, C/N ratio, substrate) influencing NTM diversity and distribution; (2) evaluate the role of aquatic macro‐biodiversity in shaping NTM patterns; (3) compare NTM diversity and distribution across continents and (4) examine correlations networks between mycobacterial species.

## 2 Materials and Methods

### 2.1 Study Sites

Figure 1 shows the location of sampling sites (Figure 1A). In French Guiana (FG), 13 aquatic sites (ponds and oxbows) were sampled along the coast in mid‐July 2017, corresponding to the end of the rainy season in FG. These included 8 urban and 5 rural sites (Table 1; Figure 1B and Figure S1A–D; maps were generated using QGIS v3.36.2). Sampling in Côte d'Ivoire (CI) occurred in May 2018, corresponding to the beginning of the rainy season in CI, and included 12 urban or peri‐urban water bodies (none of the sites could be characterised as truly rural in CI) near Bouaflé, Sinfra and Yamoussoukro (Figure 1C and Figure S2A–D). The rationale for selecting the sampling period in each country was based on: (1) the necessity for the selected aquatic ecosystems to be sufficiently supplied with water, we thus had to sample during the rainy season; (2) despite the necessity of rainfall, we had to avoid flooding which dilutes DNA; (3) sampling had to occur when our teams had planned field missions with local partners in each country. All sites were located in Buruli ulcer‐endemic areas and were selected because clinically confirmed cases had been diagnosed there by physicians from the National Buruli Ulcer Control Program (PNLUB). Additionally, *M. ulcerans* had previously been detected at these sites, both in water bodies (Combe et al. 2019) and on plants (Hammoudi et al. 2020).

**FIGURE 1:**
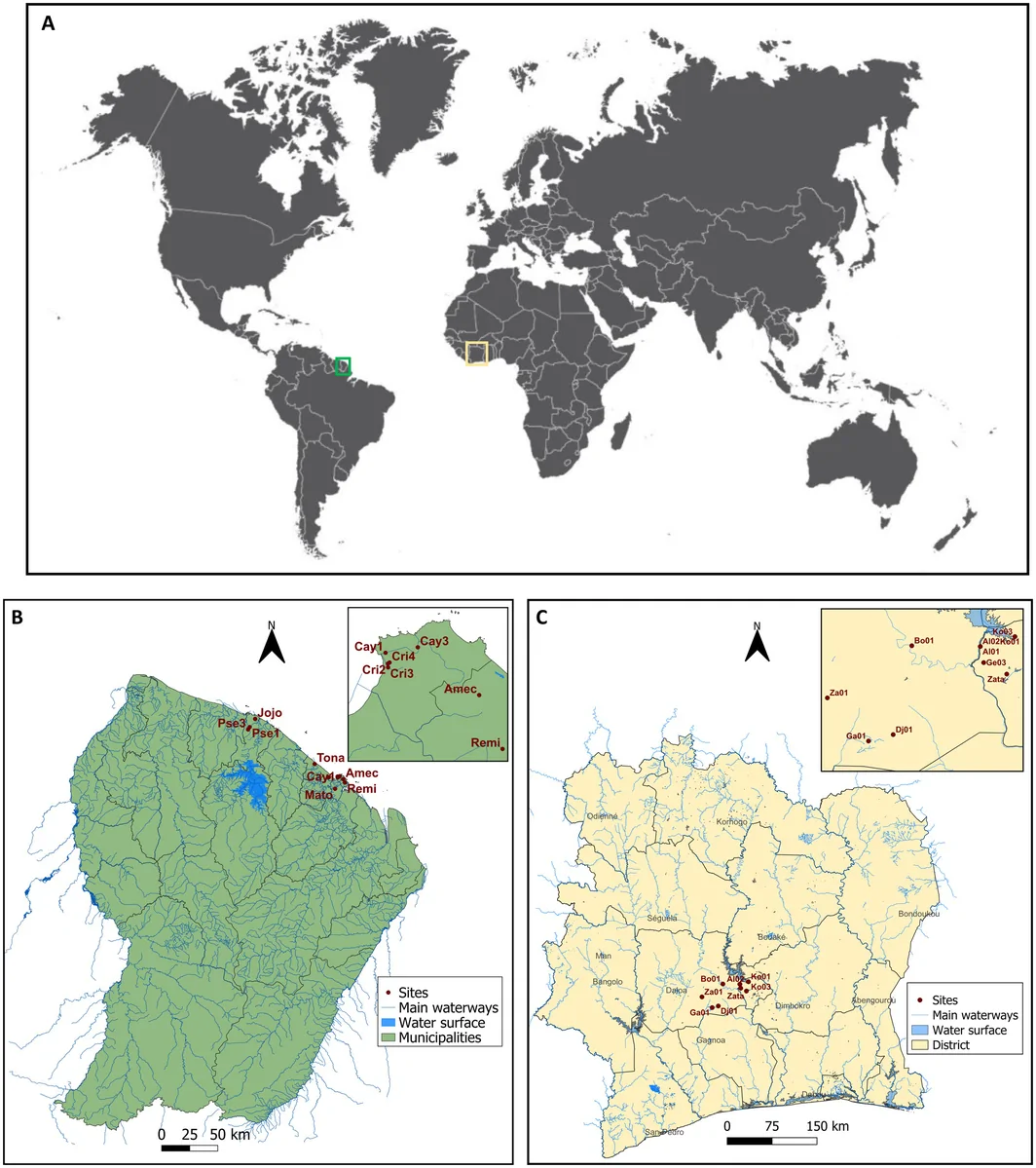
Worldwide map with the localisation of French Guiana (FG) and Côte d'Ivoire (CI) (A). Localisation of sampling sites in French Guiana (B) and Côte d'Ivoire (C). Sampling sites are indicated in red (see Table 1 for site abbreviations).

**TABLE 1:** Study sites in French Guiana (FG) and Côte d'Ivoire (CI).

Country	Site name	Abbreviation	Latitude	Longitude	Area	Samples
FG	Jojo	Jojo	5.3941	−52.992017	Rural	W, SS
FG	Piste St Elie 1	Pse1	5.32946	−53.03592	Rural	W, SS
FG	Piste St Elie 3	Pse3	5.31043	−53.04884	Rural	W, SS
FG	Tonate	Tona	5.03535	−52.516483	Rural	W, SS
FG	Matoury	Mato	4.838083	−52.35325	Rural	W, SS
FG	Soula	Soul	4.929067	−52.403817	Urban	W, SS
FG	Cayenne 1	Cay1	4.93505	−52.32441	Urban	W, SS
FG	Cayenne 3	Cay3	4.93786	−52.31704	Urban	W, SS, SC
FG	Crique 2	Cri2	4.92908	−52.33273	Urban	W, SS, SC
FG	Crique 3	Cri3	4.92697	−52.33201	Urban	W, SS
FG	Crique 4	Cri4	4.93037	−52.33123	Urban	W, SS
FG	Ame Claire	Amec	4.91411	−52.2864	Urban	W, SS, SC
FG	Remire	Remi	4.88733	−52.27473	Urban	W, SS, SC
CI	Zagueta	Za01	6,783342	−6.067334	Urban	W, SS
CI	Bouaflé	Bo01	6,977789	−5.752347	Urban	W, SS
CI	Djenedougoufla	Dj01	6,6465	−5.821809	Urban	W, SS
CI	Gbalibonou	Ga01	6.622534	−5.913820	Urban	W, SS
CI	Zatta	Zata	6.872015	−5.398372	Urban	W, SS
CI	Geoffroy, Toumobokro	Ge02	6.914796	−5.484185	Urban	W, SS
CI	Geoffroy, Toumobokro	Ge03	6.914796	−5.484185	Urban	W, SS
CI	Allaiyaokro/Allanykro	Al01	6.974977	−5.497005	Urban	W, SS
CI	Allaiyaokro/Allanykro	Al02	6.974977	−5.497005	Urban	W, SS
CI	Kongouanou	Ko03	7.011667	−5.367778	Urban	W, SS
CI	Kongouanou	Ko01	7.011667	−5.367778	Urban	W, SS
CI	NA	Vo02	NA	NA	Urban	W, SS

Abbreviations: NA, unknown information; SC, sediment core samples; SS, surface sediment samples; W, water samples.

### 2.2 Environmental Sampling

In French Guiana, sampling was carried out under the French Nagoya Protocol (#166344). In Côte d'Ivoire, sampling was carried out after submitting a request for authorisation under the Nagoya Protocol to the Ministry, and in consultation with local collaborators from the National Buruli Ulcer Control Program (PNLUB). At each site, 20 × 100 mL water samples were collected from different micro‐habitats (total of 2 L) using a sterile ladle and were pooled into 2 L Whirl‐Pak bags (thus the 20 ladle samples finally form a single 2 L sample). Water was immediately filtered through 0.45 μm VigiDNA filter capsules (SPYGEN, France) using a sterile syringe until depletion or clogging. The use of such 0.45 μm VigiDNA filter was previously validated by.

SPYGEN, and is now commonly used in eDNA assays since most DNA present in the water column will be adsorbed onto organic matter and other particles (Cantera et al. 2019; Elliott et al. 2026). Capsules were filled with 80 mL CL1 conservation buffer (SPYGEN, France) and stored at room temperature before DNA extraction. Physicochemical parameters (temperature, oxygen, pH, conductivity) were recorded (Table S1). Surface sediments (0–1 cm) were sampled at all sites using a sterile Falcon tube and stored at 4°C for 24 h before DNA extraction. Additionally, sediment cores from four urban sites in FG were collected at depths of 0–1 cm; 1–2 cm; 2–3 cm; 3–4 cm; 4–5 cm; 5–10 cm and subsampled at each sediment layer for DNA extractions and physicochemical analysis (P, N, C, C/N ratio, granulometry) (Table S1).

### 2.3 Aquatic Invertebrate Sampling

Invertebrates were collected using a 25 cm × 45 cm dip net (500 μm mesh) from five areas per site, sampling across microhabitats. The net was moved from the sediment surface upward over a 1m^2^ area. Specimens were sorted and preserved in 70% alcohol for taxonomic identification at the family level (Table S2).

### 2.4 DNA Extractions

For water, the DNA extraction was performed following the protocol described in (Pont et al. 2018). Each filtration capsule was agitated for 15 min on S50 Shaker (Cat Ingenieurbüro) at 800 rpm, and the CL1 buffer inside the capsule was emptied into a 50 mL tube that was then centrifuged for 15 min at 15,000 × *g*. The supernatant was removed with a sterile pipette by leaving 15 mL of liquid at the bottom of the tube. Subsequently, 33 mL of ethanol and 1.5 mL of 3 M sodium acetate were added to each 50 mL tube that were stored overnight at −20°C. The tubes were centrifuged at 15,000 × g for 15 min at 6°C, and the supernatant was discarded. Then, 720 μL of ATL Buffer from the DNeasy Blood & Tissue Extraction Kit (QIAGEN GmbH, Hilden, Germany) were added, the tubes were vortexed, and the supernatants were transferred into 2 mL tubes previously filled with 20 μL of Proteinase K. Tubes were incubated at 56°C for 2 h. Subsequently, DNA extractions were performed using the NucleoSpin Soil kit (MACHEREY‐NAGEL GmbH & Co., Düren, Germany) starting from Step 6 and following the manufacturer's instructions. Elution was performed by adding 100 μL of SE buffer twice. Negative extraction controls were included in each batch of extraction to monitor any possible contamination. Total DNA was also extracted from 250 mg of sediments using the PowerSoil DNA extraction kit (QIAGEN) and following the manufacturer's recommendations, including a negative control as well. Extracted DNA was stored at −20°C.

### 2.5 Detection of MPM by qPCR

To detect and quantify the relative abundance of MPM DNA in sediment samples we used the classical two TaqMan qPCR runs targeting the insertion sequence *IS*2404 and the ketoreductase B (KR) domain of the mycolactone polyketide synthase gene (Combe et al. 2023). To amplify *IS*2404 genetic marker, we used the following primer and probes: *IS*2404 forward primer 5′‐ATTGGTGCCGATCGAGTTG‐3′, *IS*2404 reverse primer 5′‐TCGCTTTGGCGCGTAAA‐3′ and *IS*2404 probe FAM‐CACCACGCAGCATTCTTGCCGT‐BHQ1. For KR amplification we used KR forward primer 5′‐TCACGGCCTGCGATATCA‐3′, KR reverse primer 5′‐TTGTGTGGGCACTGAATTGAC‐3′, and KR probe FAM‐ACCCCGAAGCACTG‐MGBNFQ. The qPCR reaction consisted of 1X TaqMan Gene Expression Master Mix (LifeTechnologies), 0.3 μM (final concentration) of each primer, 0.1 μM (final concentration) of the probe, 5 μL of DNA and water adjusted to a final volume per reaction of 25 μL. For KR we followed the same protocol except that we used the probe at a final concentration of 0.25 μM. An internal positive control (IPC) was added in each *IS*2404 reaction in order to test for the presence of PCR inhibitors in the environmental samples. As positive controls we used the *M. ulcerans* DNA at a concentration of 10^5^ bacteria/mL and negative (DNA replaced by sterile water) controls were included in each run. *M. ulcerans* positive control consisted of genomic DNA purified from a cultured strain from French Guiana (strain 1G897) and provided by Dr. Laurent Marsollier (ATOMYCA, Université d'Angers, France). This positive control was also used to run standard curves based on serial dilutions of purified DNA from 10^5^ to 10^0^ bacteria/mL (in triplicates). Standard curves allowed us to determine a threshold value to consider positive results (CT‐values < 38). The assays were run in an Applied Biosystems 7300 Real Time PCR system, with the following program: one cycle at 50°C for 2 min, one cycle at 95°C for 10 min, followed by 45 cycles at 95°C for 15 s and at 60°C for 1 min. Only samples with cycle threshold values < 38 for both *IS*2404 and KR markers were considered as positives (Combe et al. 2019). In all assays the negative controls remained negative.

### 2.6 Primer Design and In Silico Validation

A total of 22 group‐specific primers were designed using the *ecoprimers* software (Riaz et al. 2011) on a collection of 16S ribosomal DNA (rDNA) sequences, the most commonly used indicator gene for bacterial biodiversity assessment (Horner‐Devine et al. 2004), of the order Corynebacteriales (including all sequences present on GenBank on the 25th of August 2017). Then, we selected primer pairs with a difference of Tm < 4°C, a GC percentage comprising between 40% and 80%, indicated as GG in the output file, with a fragment length < 200 bp in order to capture degraded and fragmented DNA (as usual for eDNA studies), a Barcoding coverage (*Bc*, Ficetola et al. 2010) and Barcoding specificity (*Bs*, Ficetola et al. 2010) > 0.95 and with < 5 repetitions of the same base. The primers that passed these criteria and were retained were aligned to *M. tuberculosis* 16S and their positions on the sequence were used to determine the cluster of primers that bind a similar genomic region using the function *Mclust* in the R package Mclust. Then, one primer pair per cluster (22 clusters) was randomly chosen and tested in silico using the ecoPCR program (Bellemain et al. 2010; Ficetola et al. 2010) against the entire set of DNA sequences available from the EMBL‐European Nucleotide Archive (release 132, standard sequences) and with a maximum of three mismatches in the entire primer sequence. For each primer, a sequence logo was generated (Crooks et al. 2004) based on in silico PCR results for the target and non‐target group. A mismatch analysis was performed, both for the target and for the non‐target taxonomic group to assess specificity. Five other primer pairs available in the literature and previously used for the detection of *Mycobacterium* sp. (Kox et al. 1995; Tobler et al. 2006; Phung et al. 2013; Pontiroli et al. 2013; Radomski et al. 2013) were tested using the same protocol. Finally, one primer pair (MycoF: 5′‐CCACACCGCAAAAGCTTT‐3′; MycoR: 5′‐CGTGCTTAACACATGCAA‐3′) was selected from the newly designed ones since it was the most conserved among the *Mycobacterium* group, it amplified a low number of non‐target groups, and it amplified a fragment showing the smallest length polymorphism among the 27 primer pairs tested.

### 2.7 DNA Amplification and Sequencing

DNA amplifications were performed in a final volume of 25 μL, using 3 μL of DNA extract as template. The amplification mixture consisted of 1 U of AmpliTaq Gold DNA Polymerase (Applied Biosystems, Foster City, CA), 10 mM of Tris–HCl, 50 mM of KCl, 2.5 mM of MgCl2, 0.2 mM of each dNTP, 0.2 μM of each primer, and 0.2 μg/μL of bovine serum albumin (BSA, Roche Diagnostic, Basel, Switzerland). Primers were 5′‐labelled with an eight‐nucleotide unique tag (with at least three differences between any pair of tags) allowing the assignment of each sequence to the corresponding sample during sequence analysis. Tags for forward and reverse primers were identical for each sample. The PCR mixture was denatured at 95°C for 10 min, followed by 40 cycles of 30 s at 95°C, 30 s at 55°C and 1 min at 72°C, and followed by a final elongation at 72°C for 7 min. The PCR was performed in a room dedicated to amplify DNA, with negative air pressure and physically separated from the DNA extraction rooms (with positive air pressure). Four PCR replicates were run per sample. One negative PCR (ultrapure water, with 12 replicates), two positive controls composed of pooled DNA extracts of seven *Mycobacterium* species DNA ( *M. ulcerans* , *M. marinum* , *M. fortuitum* , *M. avium* , *M. chelonae* , *M. absessus*, *M. smegmatis* ; *M. leprae* ; *M. lepromatosis* ; Table S3) provided by Dr. Jean‐Christophe Avarre (UMR ISEM, Montpellier, France) and three negative controls (DNA replaced by sterile water) for extractions were analysed in parallel to monitor any possible contaminations and the sensitivity of the primer pairs.

Amplified DNAs were quantified using capillary electrophoresis (QIAxcel; QIAGEN) and purified using the MinElute PCR purification kit (QIAGEN). Purified PCR products were then pooled in equal volumes to achieve an expected theoretical sequencing depth of 700,000 reads per sample. Library preparation and sequencing were performed at Fasteris (Geneva, Switzerland). Libraries were prepared following the MetaFast protocol (Fateris, https://www.fasteris.com/dna/?q=content/metafast‐protocol‐amplicon‐metagenomic‐analysis) and a paired‐end sequencing (2 × 125 bp) was carried out using an Illumina MiSeq (Illumina, San Diego, CA, USA) with the MiSeq Kit v3 (Illumina, San Diego, CA, USA) following the manufacturer's instructions.

### 2.8 Bioinformatics

Sequence reads were analyzed using the programs implemented in the OBITools package (http://metabarcoding.org/obitools; Boyer et al. 2016). Forward and reverse reads were assembled using the illuminapairedend program and assigned to each sample using the ngsfilter program. Sequences shorter than 20 bp, occurring < 10 times per sample or labelled as ‘internal’ by the obiclean program (corresponding most likely to PCR/sequencing errors) were discarded. Molecular Operational Taxonomic Units (MOTUs) represent clusters of organisms grouped based on molecular similarity (DNA sequences here) rather than traditional morphological or ecological traits. They are a molecular equivalent of species or taxonomic groups, defined using genetic data. MOTUs are revolutionising the fields of ecology, microbiology, and biodiversity studies by enabling rapid, scalable, and standardised species identification and community analysis. The taxonomic assignment of MOTUs was performed using the program ecotag, with a reference database built by using the 7753 complete genomes of *Mycobacterium* sp. (available on GenBank on the 29th of September 2017, Table S4), and resulting in a total of 42 MOTUs. MOTUs showing < 97% similarity with our reference database were removed since this 97% threshold is commonly used to define bacterial species (Stackebrandt and Goebel 1994). Finally, all sequences with a frequency of occurrence below 0.002 per taxon were discarded. These thresholds were empirically determined to avoid tag‐jumps between samples (Schnell et al. 2015).

## 3 Analysis of Mycobacteria Communities' Diversity and Spatial Variability

Statistics were performed in RStudio (v2023.12.1). NA values in sequencing data, indicating absent MOTUs, were converted to zero. Genetic sequences and invertebrate taxonomic data were log10(*x* + 1) transformed to improve visualisation and meet statistical assumptions. Group differences were assessed via Student's *t*‐test (normal data) or Wilcoxon Mann Whitney test (non‐normal data) with a 5% alpha threshold. Post hoc corrections (Tukey or the Dunn's test with a Benjamini–Hochberg adjustment) were applied, when necessary, with a Kruskal–Wallis test.

### 3.1 Alpha Diversity Analysis (Vegan Package Version 2.6.4)

Alpha diversity was assessed using the Shannon index (using the function ‘diversity()’ which expects counts by default) to quantify the diversity of MOTU communities at each site. This index accounts for both species richness and evenness, providing a comprehensive measure of community structure. The analysis helps determine whether some sites host more diverse or more evenly distributed MOTU assemblages compared to others.

### 3.2 Beta Dissimilarity Analysis (Vegan Package Version 2.6.4)

To compare the composition of MOTU communities between sites, beta dissimilarity was evaluated using the Jaccard index. This measure captures differences in MOTUs presence or absence, allowing the identification of compositional variations across sampling locations. Such an approach is crucial for understanding spatial heterogeneity in microbial communities. We performed PERMANOVA to compare mycobacterial MOTU composition between and within zones (urban, rural) and between water and sediment samples.

### 3.3 MOTU Distribution in Sediment Cores

To characterise the vertical distribution of NTM MOTUs within sediment cores, an ANOVA was performed on MOTU richness (i.e., the number of distinct MOTUs per site/layer). This statistical test determines whether significant differences exist in MOTU diversity across sediment layers, helping to assess stratification patterns and potential environmental influences on microbial distribution.

### 3.4 Multidimensional Scaling Analysis (Vegan Package Version 2.6.4)

Non‐Metric Multidimensional Scales (NMDS) was employed to visualise site relationships based on similarities and dissimilarities in MOTU composition. This technique reduces data dimensionality while preserving relative distances. The NMDS approach is particularly useful for representing ecological composition data and allows for highlighting trends in species distribution between sites. ‘Stress’ was used as a measure of the goodness of fit of the data ordination. It assesses how well distances in the reduced space (represented by the NMDS) correspond to distances in the original data space. A stress < 0.05 indicates excellent representation; a stress < 0.1 is considered very good; a stress < 0.2 is acceptable; and a stress > 0.3 indicates poor representation.

### 3.5 Hierarchical Clustering Analysis (HCA) and Principal Component Analysis (PCA) (hclust Function and prcomp Function of the Stats Package Version 4.3.3, Respectively)

HCA was used to classify sites based on their MOTU composition, distinguishing clusters of similar and dissimilar sites. The optimal dendrogram cut was determined using the inertia loss scree method. Additionally, PCA was performed on transformed raw data (centered but not scaled) to further reduce dataset complexity while maintaining variance structure. This approach aids in identifying dominant gradients influencing community composition.

### 3.6 Linear Discriminant Analysis (LDA) and Random Forest (RF) (Caret Package Version 6.0.94)

LDA was applied following the PCA to formally define site grouping and identify key discriminant variables. By maximising separation between predefined groups, LDA seeks to highlight the most influential features distinguishing microbial communities. A 3‐fold, 200 repetition cross‐validation ensured model robustness. To further compare patterns between FG and CI, a RF analysis was conducted leveraging its ability to produce accurate predictions and assess variable importance.

### 3.7 MOTU Co‐Occurrence Network (qgraph Package Version 1.9.8.)

Network analysis was performed to investigate potential co‐occurrence patterns among MOTUs, for each type of habitat (rural, urban, water, surface sediment, core sediment). This method helps uncover co‐occurrence patterns, which can reveal ecological interactions such as mutualistic or competitive relationships among microbial taxa. A Spearman's correlation between each MOTU, commonly used for non‐linear datasets, was calculated and correlations > 0 identified from pairwise comparisons were considered to further run and visualise a partial correlation network by using the function ‘pcor’ in the qgraph package in R (Epskamp et al. 2012), where each node represents a MOTU and each edge stands for a correlation between the nodes, assuming that the bigger the edges (lines), the stronger the link between two MOTU.

### 3.8 Mycobacteria‐Invertebrate Relationships (Coinertia Function of the ade4 Package Version 1.7.22)

To explore the relationship between mycobacteria MOTUs and invertebrate communities, a co‐inertia analysis was conducted. This technique identifies covariation patterns between two datasets, providing insights into potential ecological interactions. It is particularly useful for understanding how microbial diversity correlates with macro‐organism distributions, shedding light on functional linkages in the studied environment.

## 4 Results

Illumina MiSeq paired‐end sequencing yielded 73,881,081 reads, with 21,497,222 reads (298,573 per sample on average) assigned to 39 mycobacterial MOTUs at 98% similarity threshold, including an unidentified *Mycobacterium* sp. (Myco) MOTU. Whilst we focused only on NTM, Myco_group4 (Gp04) also encompassed *M. tuberculosis* due to its shared 16S region sequence targeted in this study with other MOTUs (Table 2). In FG, sequencing from water and sediments produced 36,800,559 reads, with 12,245,891 (510,246 per sample on average) assigned to MOTUs (Figure S3A), while sediment cores generated 19,919,577 reads, with 5,767,468 (240,311 per sample on average) assigned (Figure S3B). In CI, 17,160,945 reads were obtained, with 3,483,863 (145,161 per sample on average) assigned to MOTUs (Figure S3C). Also, a *M. lepromatosis* MOTU, a causative agent of leprosy, was included to assess its presence in environmental samples, but was not detected at all. A total of 27 MOTUs were found in both countries while some were only found country‐specific. In FG, unique MOTUS (*N* = 11) included *M. phlei* (Phle), *M. liflandii* (Lifl), *M. immunogenum* (Immu), *M. goodi* (Good), *M. avium* (Aviu), *M. avium* complex (Gp13), Myco‐Group8 (Gp08 including *M. marinum* / *M. ulcerans* ), Myco‐Group7 (Gp07 including *M. sinense* / *M. terrae* ), Myco_Group4 (Gp04 including *M. africanum* /*M. bovis/M. canettii/M. caprae/M. tuberculosis*), Myco_Group3 (Gp03 including *M. absessus* /*M. chelonae/M. saopaulense/M*. sp. *QIA‐37/M. stephanolepidis*) and Myco_Goup10 (Gp10 including *M. chimaera/M. indicus pranii/M. intracellulare/M*. sp. *MOTT36Y/M. yongonense*), whereas *M. aurum* (Auru) was exclusive to CI (Table 2 and Figure S4).

**TABLE 2:** Dataset of the mycobacterial MOTUs identified by eDNA analyses of water and sediments (surface and core) in French Guiana (FG) and Côte d'Ivoire (CI).

MOTU	Abbreviation	Mycobacterial species (16S)	Country
Myco_group1	Gp01	*Mycobacterium marseillense* , *Mycobacterium yongonense*	FG, CI
Myco_group10	Gp10	*Mycobacterium chimaera* , *Mycobacterium indicus pranii* , *Mycobacterium intracellulare* , *Mycobacterium* sp. *MOTT36Y*, *Mycobacterium yongonense*	FG
Myco_group11	Gp11	*Mycobacterium* sp. *KMS*, *Mycobacterium* sp. *MCS*	FG, CI
Myco_group12	Gp12	*Mycobacterium* sp. *JLS*, *Mycobacterium* sp. *KMS*	FG, CI
Myco_group2	Gp02	*Mycobacterium vaccae* , *Mycobacterium vanbaalenii*	FG, CI
Myco_group3	Gp03	*Mycobacterium abscessus* , *Mycobacterium chelonae* , *Mycobacterium saopaulense* , *Mycobacterium* sp. *QIA‐37*, *Mycobacterium stephanolepidis*	FG
Myco_group4	Gp04	*Mycobacterium africanum* , *Mycobacterium bovis* , *Mycobacterium canettii* , *Mycobacterium caprae* , *Mycobacterium tuberculosis*	FG
Myco_group5	Gp05	*Mycobacterium pallens* , *Mycobacterium* sp. *WY10*	FG, CI
Myco_group6	Gp06	*Mycobacterium fortuitum* , *Mycobacterium* sp. *VKM Ac‐1817D*	FG, CI
Myco_group7	Gp07	*Mycobacterium sinense* , *Mycobacterium terrae*	FG
Myco_group8	Gp08	*Mycobacterium marinum* , *Mycobacterium ulcerans*	FG
Myco_group9	Gp09	*Mycobacterium neonarum* , *Mycobacterium* sp. *NRRL B‐3805*	FG, CI
*Mycobacterium*	Myco	*Mycobacterium* sp.	FG, CI
*M. aurum*	Auru	*Mycobacterium aurum*	CI
*M. avium*	Aviu	*Mycobacterium avium*	FG
*M. avium complex* (*MAC*)	Gp13	*Mycobacterium avium complex* (*MAC*)	FG
*M. chubuense*	Chub	*Mycobacterium chubuense*	FG, CI
*M. colombiense*	Colo	*Mycobacterium colombiense*	FG, CI
*M. dioxanotrophicus*	Diox	*Mycobacterium dioxanotrophicus*	FG, CI
*M. gilvum*	Gilv	*Mycobacterium gilvum*	FG, CI
*M. goodii*	Good	*Mycobacterium goodii*	FG
*M. haemophilum*	Haem	*Mycobacterium haemophilum*	FG, CI
*M. immunogenum*	Immu	*Mycobacterium immunogenum*	FG
*M. kansasii*	Kans	*Mycobacterium kansasii*	FG, CI
*M. lepromatosis*	Lepr	*Mycobacterium lepromatosis*	Not found
*M. liflandii*	Lifl	*Mycobacterium liflandii*	FG
*M. litorale*	Lito	*Mycobacterium litorale*	FG, CI
*M. pallens*	Pall	*Mycobacterium pallens*	FG, CI
*M. phlei*	Phle	*Mycobacterium phlei*	FG
*M. rhodesiae*	Rhod	*Mycobacterium rhodesiae*	FG, CI
*M. rutilum*	Ruti	*Mycobacterium rutilum*	FG, CI
*M. shigaense*	Shig	*Mycobacterium shigaense*	FG, CI
*M. simiae*	Simi	*Mycobacterium simiae*	FG, CI
*M. smegmatis str. MC2 155*	Smeg	*Mycobacterium smegmatis str. MC2 155*	FG, CI
*Mycobacterium* sp. *djl‐10*	Dj10	*Mycobacterium* sp. *djl‐10*	FG, CI
*Mycobacterium* sp. *EPa45*	Epa4	*Mycobacterium* sp. *EPa45*	FG, CI
*Mycobacterium* sp. *JS623*	Js62	*Mycobacterium* sp. *JS623*	FG, CI
*Mycobacterium* sp. *MS1601*	Ms16	*Mycobacterium* sp. *MS1601*	FG, CI
*Mycobacterium* sp. *YC‐RL4*	Ycrl	*Mycobacterium* sp. *YC‐RL4*	FG, CI
*Mycobacterium vanbaalenii*	Vanb	*Mycobacterium vanbaalenii*	FG, CI

*Note:* The name and the abbreviation of each MOTU are indicated, as well as the different mycobacterial species belonging to each MOTU based on their 16S genetic sequence.

### 4.1 Diversity and Distribution of Mycobacterial MOTUs in FG Freshwater Habitats

#### 4.1.1 Urban vs. Rural Sites

Hierarchical Cluster Analysis (HCA) completed with PERMANOVA were used to test for beta diversity (diversity between sites) using the Jaccard distance as the dissimilarity index and average as the clustering method. HCA grouped sites into urban and rural clusters (Figure 2A) and PERMANOVA showed a highly significant effect (*F* = 6.18; *p* = 0.0005, *n* = 24) of the urban/rural zone factor which explained 21.9% of the total compositional variance (*R* ^2^ = 0.219). Together, these results suggested that community composition differed significantly between rural and urban zones (the test for homogeneity of multivariate dispersions (*betadisper*) revealed no significant difference in dispersion between the two zones (*F* = 1.869; *p* = 0.185) and the distribution of samples between the two zones was relatively balanced (rural *n* = 10; urban *n* = 14)).

**FIGURE 2:**
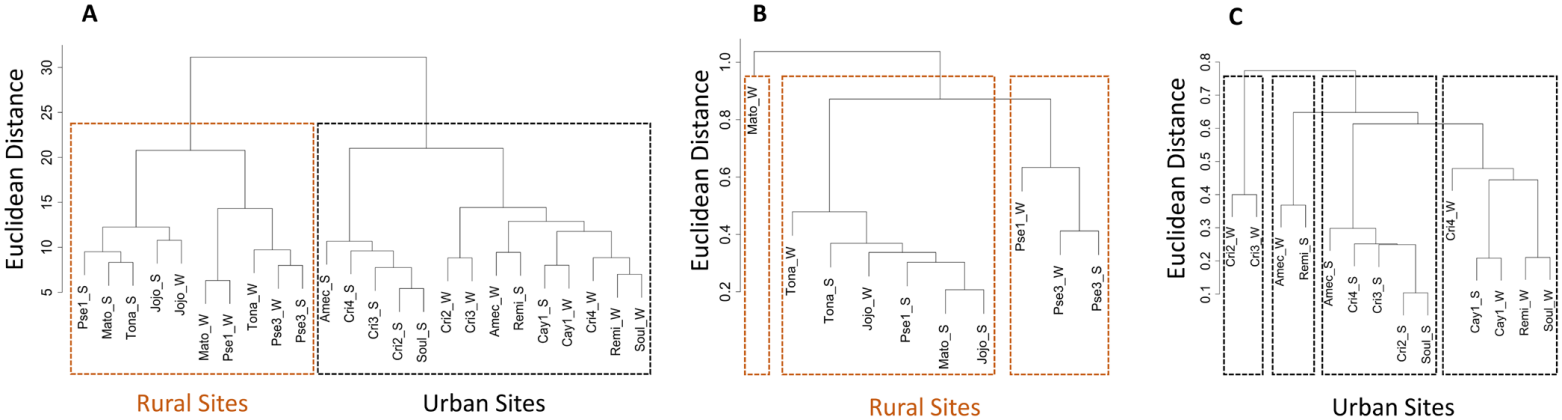
(A) Hierarchical Cluster Analysis (HCA) of log‐transformed data (reads) collected in French Guiana (FG). (B, C) HCA of the dissimilarity matrix (Jaccard index). We used the Euclidean distance and the wardD2 jump. Site names are indicated (see Table 1 for site abbreviations) as well as the type of sample collected such as water (W) or surface sediment (S). (B) Rural sites; (C) urban sites.

HCA further identified subgroups based on sample type (water vs. sediment; Figure 2A). However, some sites deviated from this pattern, such as Jojo water (Jojo_W) clustering with sediment samples, or Piste St Elie 3 sediment (Pse3_S) clustering with water samples. Similarly, urban sites Remire sediment (Remi_S) and Cayenne 1 sediments (Cay1_S) clustered with urban water samples. In line with this, the PERMANOVA showed that the water/sediment segregation explained 11.5%, suggesting a significant community composition between water and sediment samples (*F* = 2.86; *p* = 0.008, *n* = 24). Moreover, the distribution of samples between the two compartments was perfectly balanced (*n* = 12 for sediment; *n* = 12 for water), which reinforces the reliability and robustness of the statistical tests performed. However, the test for homogeneity of multivariate dispersions also revealed a significant difference in dispersion between the two compartments (*F* = 7.94; *p* = 0.010). Such heterogeneity of variances represents a significant limitation for interpreting the PERMANOVA result, since the test simultaneously assesses differences in location (centroids) and dispersion, meaning the observed significant effect (*p* = 0.008) could reflect—at least in part—this difference in dispersion between water and sediment, rather than a strict difference in mean community composition between the two compartments.

However, Principal Component Analysis (PCA) revealed distinct MOTU compositions between urban and rural sites (Figure 3A). *M. shigaense* (Shig), *M. haemophilum* (Haem), and Myco_goup4 (Gp04) were predominantly associated with rural sites, while Myco_group6 (Gp06), *M. vanbaaleni* (Vanb) and *M. avium complex* (Gp13) were predominantly found in urban environments (Figure 3B). Additionally, urban sites showed a segregation between water and surface sediment samples (Figure 3A). Linear Discriminant Analysis (LDA) achieved 89% classification accuracy, identifying *M. shigaense* (Shig), Myco_group 6 (Gp06), and *M. haemophilum* (Haem) as key discriminants between urban and rural sites (Figure S5). The 100% site specificity of *M. shigaense* (rural) and Myco_group6 (urban) suggest that their distribution is strongly linked to site specific micro‐characteristics.

**FIGURE 3:**
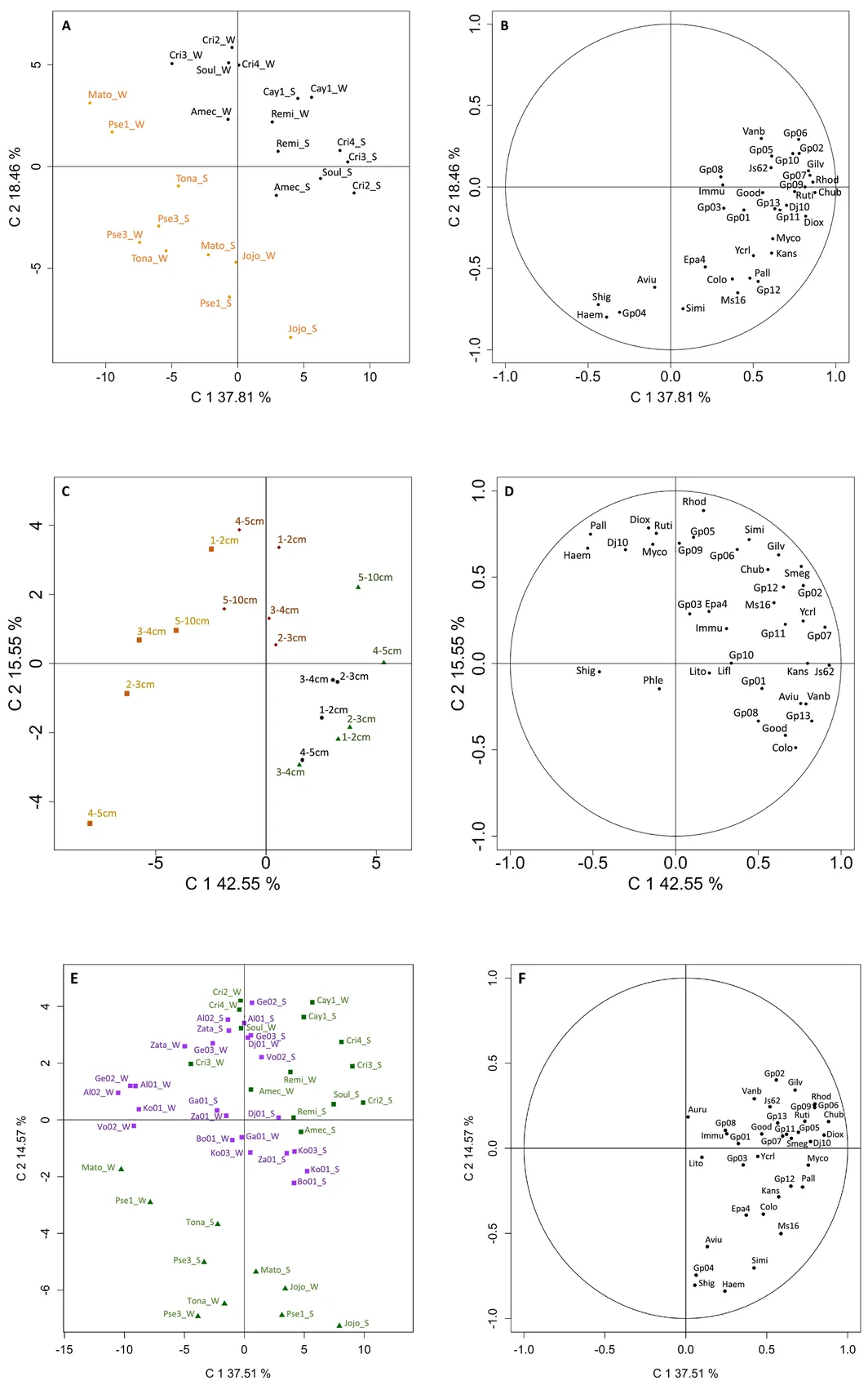
(A, B) Principal component analysis (PCA) on log‐transformed read data. Axis 1 explains 37.81% of the variability of the dataset while Axis 2 explains 18.46% of this variability. (A) Cloud of the sites. Rural sites are indicated in orange while urban sites are indicated in black; (B) Circle of correlations between MOTUs and axes. The name of each site and each MOTU is indicated (see Tables 1 and 2). S: Surface sediment samples; W: Water samples. (C, D) Unreduced centered PCA on log10(*x* + 1) transformed core data. The Cay3_5–10 cm value was excluded as it was not representative. All sediment layers for the four sites (1–2 cm, 2–3 cm, 3–4 cm, 4–5 cm, 5–10 cm) are represented. (C) Cloud of individuals (sites); Orange square = Amec, Brown diamond = Remi, Green triangle = Cri2, Black circle = Cay3. (D) Circle of correlations between MOTUs and components. Axis C1 explains 42.55% of the variability of the dataset and axis C2 15.55%. (E, F) Unreduced centered PCA on log10(*x* + 1) transformed French Guiana (FG) and Côte d'Ivoire (CI) MOTU data. C1 explains 37.51% of the variability in the dataset and C2 14.57%. (E) Cloud of individuals (sites); (F) circle of correlations between MOTUs and components. The sites were coloured according to the country (FG in green and CI in purple), the triangles represent sites in rural areas and the squares in urban areas.

Given the significant urban versus rural clustering, we further analysed MOTU composition separately for each. HCA (Ward's method, Jaccard dissimilarity index) identified three rural site groups within the rural samples (Figure 2B). To assess whether this structuring corresponds to a significant differentiation in community composition, a PERMANOVA was performed on the Jaccard distance matrix and showed that 20.6% of the total compositional variance (*R* ^2^ = 0.206) was explained by this grouping. However, this effect did not reach the conventional significance threshold (*F* = 2.07; *p* = 0.083, *n* = 10). These results suggest a trend towards differentiation among the three identified groups, without, however, allowing for a definitive conclusion regarding a difference in community composition—likely due to the small sample size (*n* = 10 distributed across three groups), which limits the statistical power of the test. Moreover, we observed that sites Piste St Elie 1 (Pse1) and Piste St Elie 3 (Pse3) clustered due to their upstream‐downstream proximity, but Piste St Elie 1 sediment (Pse1_S) diverged, suggesting again that mycobacterial diversity might be better influenced by specific microhabitat conditions than watershed dynamics. Similarly, Matoury water (Mato_W) clustered separately, indicating that local abiotic/biotic factors may shape mycobacterial distribution.

For urban sites, HCA identified four distinct clusters (Figure 2C), with Crique 4 water (Cri4_W) forming a unique group, suggesting a distinct MOTU composition compared to other urban sites in the same cluster. Whilst the NMDS showed a stress of 0.127, suggesting that the model is acceptable (Figure S6), the PERMANOVA found a highly significant effect.

(*F* = 4.49; *p* = 0.0005, *n* = 14), with 57.4% of the variance in community composition being explained by this grouping (*R* ^2^ = 0.574). Since the distribution of sites across the four groups was quite balanced (*n* = 5, 2, 5, 2), with no single‐site groups, and that the test for homogeneity of multivariate dispersions revealed no significant differences in dispersion between groups (*F* = 1.306; *p* = 0.326), the results tended towards a strong and statistically robust difference in community composition between the four urban groups.

#### 4.1.2 Water vs. Surface Sediment Samples

Alpha diversity (Shannon index) did not significantly differ between urban (U) and rural (R) sites or between water (W) and sediment (S) samples (ANOVA, *p* = 0.126; Figure 4A) nor when comparing only water versus sediment (Wilcoxon Mann–Whitney, *p* = 0.101; Figure 4B). However, median alpha diversity tended to be higher in surface sediments (R_S, U_S) than in water samples (R_W, U_W; Figure 4A). Also, the number of MOTUs was significantly higher in surface sediments than in water across all comparisons (R_S vs. R_W, U_S vs. U_W, and U_S vs. R_W; ANOVA, *p* = 0.002; Figure 4C), with a marked and significant difference between sediment and water samples (*t*‐test, *p* < 0.001; Figure 4D). These findings suggest greater mycobacterial MOTU diversity in surface sediments compared to water samples.

**FIGURE 4:**
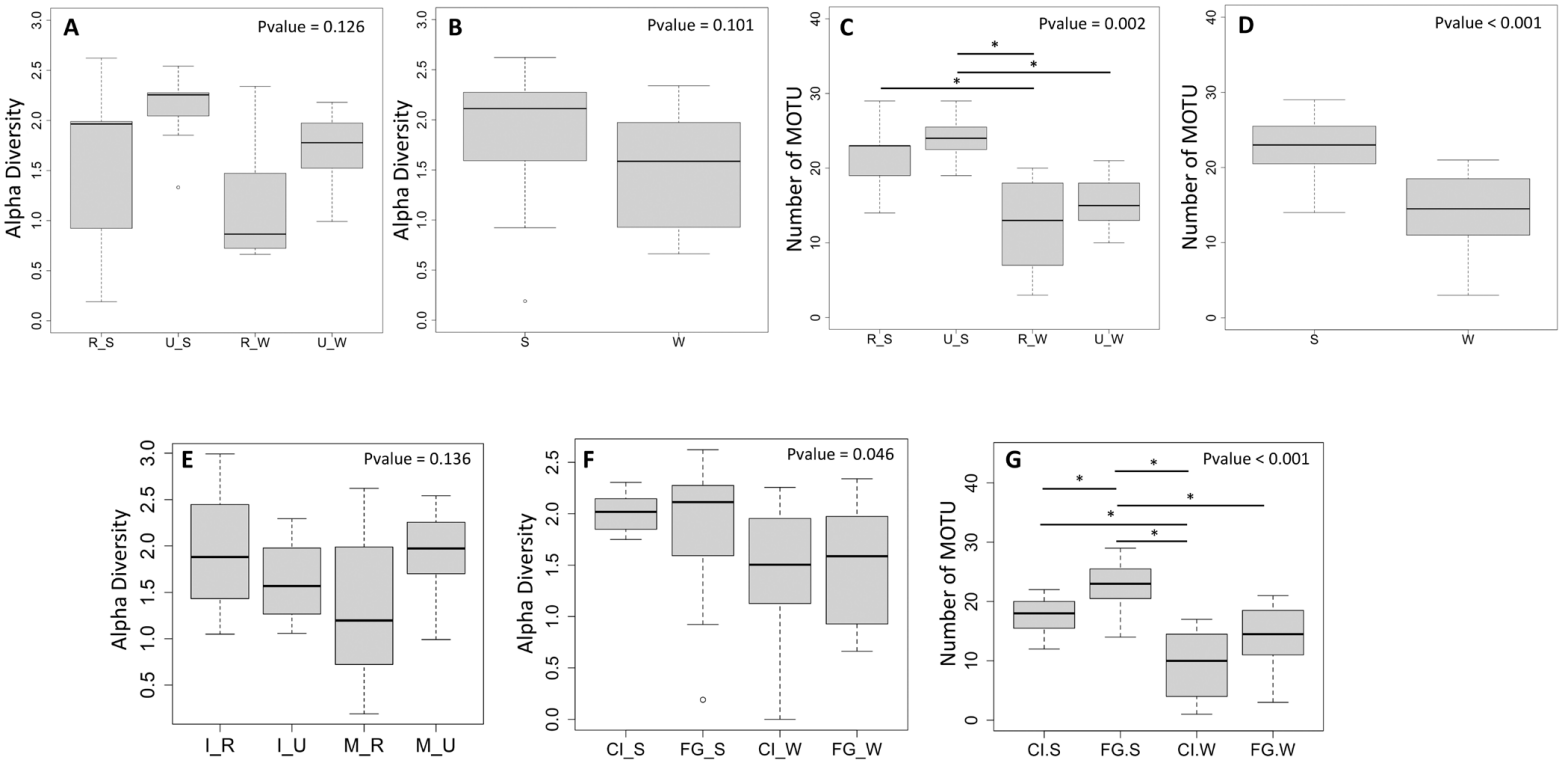
MOTU distribution and diversity in French Guiana (FG) and Côte d'Ivoire (CI). (A) Alpha (Shannon) diversity for each type of site and for each type of samples. (B) Alpha (Shannon) diversity for surface sediments and water samples. (C) Distribution of the number of MOTUs for each type of site and for each type of samples. (D) Distribution of the number of MOTUs for surface sediments and water samples. (E) Distribution of alpha diversity (Shannon index) for invertebrates (I) and mycobacteria (M) communities in rural and urban environments. (F, G) Distribution of alpha diversity (Shannon) (F) and the number of MOTUs (G) by country and by sample type. U: Urban sites; R: Rural sites; S: Surface sediment samples; W: Water samples; FG: French Guiana; CI: Côte d'Ivoire. The black bar indicates the median and the moustaches the extremum. Circles indicate extreme values, that is, values greater than 1.5 times the interquartile space and the segments with * represent significant differences.

#### 4.1.3 Vertical Distribution of Mycobacterial MOTUs

Core sediment sampling from four FG sites in FG (Amec, Remi, Cay3, Cri2) revealed no significant differences in MOTU richness across sediment depths (ANOVA, *p* = 0.744; Figure S7), despite a trend of decreasing MOTU numbers with depth. However, MOTU richness varied significantly between sites (all depths grouped together), with Amec exhibiting lower diversity than Cay3 and Cri2 (Kruskal–Wallis, *p* = 0.002; Figure S8). HCA grouped the sites into two clusters: Cay3/Cri2 and Amec/Remi (Figure S9). PCA confirmed this grouping, indicating distinct mycobacterial communities (Figure 3C). *M. pallens* (Pall) and *M. haemophilum* (Haem) were preferentially found in Amec/Remi, while the *M. avium complex* (Gp13) was more represented in Cay3/Cri2 sediment cores (Figure 3D). LDA identified Gp13, Pall, and sediment phosphorus (P) as key discriminants between site groups (accuracy = 0.87; Figure S10). MOTU Gp13 varied significantly between Amec and Cay3, Amec and Cri2, and Cri2 and Remi (ANOVA, *p* = 0.002; Figure 5A), while phosphorus levels differed between nearly all sites (ANOVA, *p* < 0.001; Figure 5B). The distribution of MOTUs Pall, Vanb, Js62, and Haem also showed significant site‐specific differences (Kruskal‐Wallis, *p* < 0.005; Figure 5C–F). Overall, the clustering of sites Cri2/Cay3 versus Amec/Remi was supported by both microbial and environmental variables, with site‐specific mycobacterial distributions likely driven by local abiotic and biotic factors.

**FIGURE 5:**
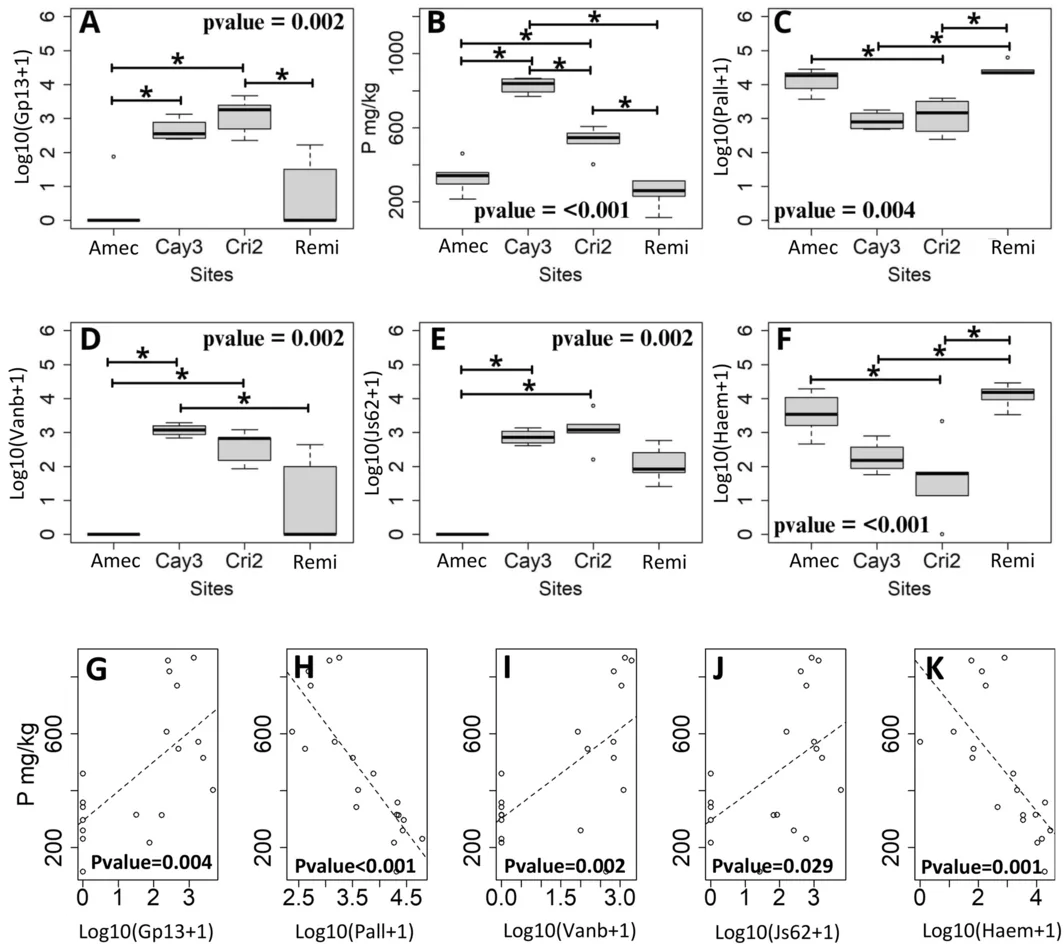
(A–F) Distribution of the MOTU Gp13, Pall, Vanb, Js62 and Haem and the phosphorus (P) according to the 4 sites sampled for sediment layers (1–2 cm, 2–3 cm, 3–4 cm, 4–5 cm, 5–10 cm). The black bar indicates the median and the whiskers the extremum. The circle indicates an extreme value, that is, greater than 1.5 times the interquartile range and the segments with ‘*’ represent significant contrasts. (G–K) Relationship between phosphorus (P) levels and different MOTUs found in the sediment layers (1–2 cm, 2–3 cm, 3–4 cm, 4–5 cm, 5–10 cm) of four aquatic sites (Amec, Remi, Cri2, Cay3) analysed in French Guiana (FG). Here, log10(*x* + 1) was used to avoid the logarithm of 0 which is undefined and thus to improve visualisation. Gp13: *M. avium complex* (*MAC*), Pall: *M. pallens* , Vanb: *M. vanbaalenii* , Js62: *M*. sp. *Js62*, Haem: *M. haemophilum* (see Table 2). Amec: Site Ame Claire; Cay3: Site Cayenne 3; Cri2: Sites Crique 2; Remi: Site Remire (see Table 1).

#### 4.1.4 Abiotic Drivers of Mycobacterial MOTU Distribution

To explore abiotic influences on mycobacterial MOTU distribution, we analysed key chemical parameters in urban and rural sites (including temperature, pH, oxygen, conductivity for water; C, N, C/N for sediments). Although no significant impacts were detected for most of these abiotic parameters (Wilcoxon, *p* > 0.05; Figure S11A–C), urban sites tended to exhibit higher pH (Figure S11D). However, the limited sample size warrants caution in interpretation.

Given the observed structuring of sediment‐associated mycobacteria based on phosphorus levels (Figure 5B and Figure S10), we further examined correlations between phosphorus and specific MOTUs. *M. avium* complex (Gp13), Vanb, and Js62 were positively correlated with phosphorus (*p* < 0.05; Figure 5G,I,J), whereas Pall and Haem showed negative correlations (*p* < 0.05; Figure 5H,K), suggesting phosphorus availability plays a role in shaping mycobacterial communities.

We further assessed the influence of sediment composition (0–5 cm vs. 5–10 cm) across four sites (Amec, Remi, Cay3, Cri2). Amec and Remi were dominated by coarse sands (> 75%) whereas Cay3 and Cri2 contained mostly clays and fine silts (Figure S12). Random Forest (RF) analysis identified Pall, Haem, and fine silts (F.Slt.) as key variables distinguishing Ame/Rem from Cay3/Cri2 (Figure S13A,B). MOTUs Pall and Haem were associated with coarse sand‐rich sites (Amec/Remi) but absent from fine silt/clay‐dominated sites (Cay3/Cri2; Figures S14A,B and S15). RF analysis further indicated that MOTUs Gp01, Diox, and Gilv along with fine sand (F.Sd) differentiated the 0–5 cm and 5–10 cm layers, with all being more prevalent in the upper layer (*p* < 0.05, Figures S16A,B–S18A,B,C). These results suggest a stratified distribution of mycobacterial communities, with certain MOTUs favouring specific sediment textures and depths.

#### 4.1.5 Biotic Drivers of Mycobacterial MOTU Distribution

Alpha diversity (Shannon index) did not differ significantly between rural and urban sites for either invertebrates or mycobacteria (ANOVA, *p* = 0.136; Figure 4E), suggesting comparable community diversity across FG. Consequently, we further combined rural and urban sites diversity to perform a co‐inertia analysis (CoIA) that allowed us to explore potential associations between specific mycobacterial MOTUs and aquatic invertebrate communities. CoIA revealed site‐specific patterns in microbial‐invertebrate associations. Canonical weights highlighted some key invertebrate species and mycobacterial MOTUs contributing to covariance (Figure 6A), with longer vectors indicating stronger relationships. It was for instance the case for invertebrate taxa 54 and *M. smegmatis* (Smeg), or for invertebrate taxa 38 and *M. shinguense* (Shig) (see Table 2 and Table S2). Site projection (Figure 6B) showed variable links between mycobacterial and invertebrate communities; for example, sites Remi and Pse3 exhibited strong concordance (site samples grouped on the same part of the plot), whereas site Amec showed divergence. Overall, the RV coefficient (0.33) indicated weak correlation between datasets. These findings suggest that while some sites share specific mycobacterial and invertebrate communities, there is generally little alignment between the two across French Guiana suggesting that the distribution of mycobacterial MOTUs is not dependent on the presence of specific invertebrate communities.

**FIGURE 6:**
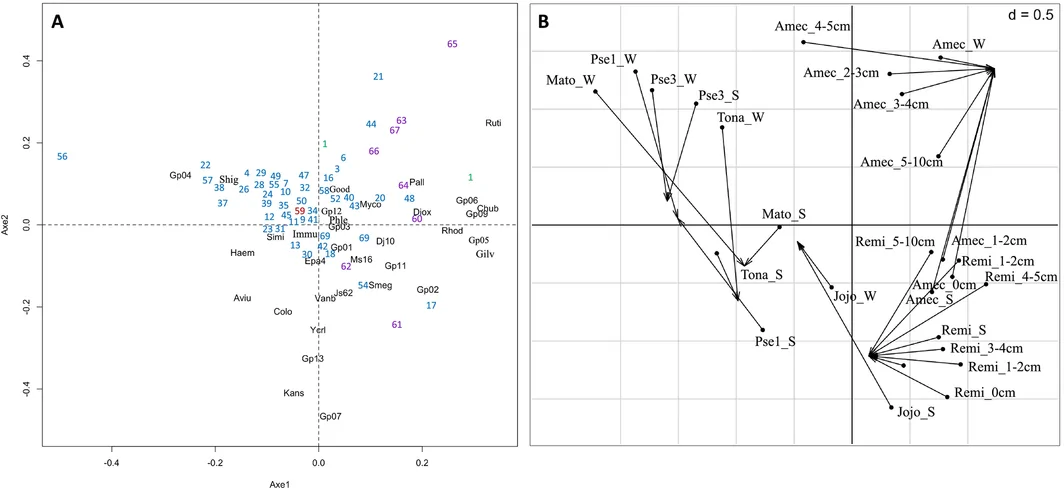
(A) Biplot of coefficients of environmental variables (Invertebrates) and MOTUs. The plot shows the canonical weights for invertebrate variables (*Y* axis) and mycobacterial MOTUs (*X* axis) on the first two axes of co‐inertia. Each invertebrate species is represented by a number (see Table S2), and the direction and distance from the origin indicate its relative influence on the axes, and each MOTU is represented in black by its abbreviation. Variables with a large length are the most important in explaining the covariance with mycobacterial variables. (B) Individual‐line cloud (sites). The starting point of the arrow represents the position of the observation in the first data set *X*, and the tip of the arrow represents its position in the second data set *Y*.

#### 4.1.6 Comparison of Mycobacterial Diversity and Distribution in FG and CI

We assessed whether mycobacterial communities differed between FG and CI. In both countries, most MOTU diversity was captured after sampling ~12 sites (Figure S19), suggesting a limited number of rare species. In FG we detected a higher number of MOTUs than in CI (ANOVA, *p* < 0.001; Figure 4G; Figures S4 and S20), with sediments harbouring more MOTUs than water samples in both regions. Alpha diversity initially showed significant differences between countries (Kruskal‐Wallis test, *p* = 0.046; Figure 4F), but post hoc tests revealed no significant contrasts. Tukey's test confirmed that mycobacterial communities varied by geography and environment type. PCA analysis showed clustering of rural and urban sites, with CI communities resembling FG urban sites, except for peri‐urban sites Ko01, Ko02, Al01, and Al02 (Figure 3E). Certain MOTUs, such as Shig, Haem, and Gp04, were associated with rural areas, while Gp02 and Gilv were linked to urban areas (Figure 3F). Random Forest analysis identified the MOTU Shig as the strongest discriminator between urban and rural sites in both countries (Figure S21). These findings suggest that some mycobacterial MOTUs are habitat‐specific, while others are more ubiquitous across FG and CI.

#### 4.1.7 Distribution of Potentially Pathogenic Mycobacteria

Our analysis identified either known pathogenic mycobacterial species for humans or MOTUs that can include pathogenic mycobacteria (Table 2 and Table S5). For instance, in FG we identified pathogenic species such as *M. immunogenum* and *M. liflandii* , but also MOTUs that can include *M. intracellulare* , *M. abscessus* , *M. chelonae* , *M. bovis* , *M. terrae* , *M. immunogenum* , *M. liflandii* , *M. marinum* , and *M. ulcerans*. Also, in both countries we found pathogenic species such as *M. gilvum* , *M. kansasii* , and *M. simiae* , while MOTU GP06 could indicate the presence of *M. fortuitum* in FG and CI. Notably, members of the Mycolactone‐Producing‐Mycobacteria (MPM) complex such as *M. marinum* , *M. ulcerans* , and *M. liflandii*, which cause Buruli ulcer (BU), were detected at low prevalence in only two FG sites and were absent in CI by eDNA analysis. This contrasts with MPM qPCR assays (*IS*2404 and KR‐B), which detected three positive sites in FG, including water and sediment samples, but a 100% positivity in CI in sediment samples, while all water samples were negative (data not shown). These discrepancies could be explained by a very low environmental abundance of MPMs, far below the detection limit of eDNA‐based metabarcoding approaches in the case of cryptic species. Additionally, *M. gilvum* —previously found associated with Buruli ulcer cases in FG—was found in both FG and CI across all habitat types (water, surface, and core sediment) at high prevalence, confirming its ubiquity in environmental matrices.

#### 4.1.8 Patterns of Mycobacterial MOTU Co‐Occurrences in FG and CI

To assess potential ecological interactions among mycobacterial MOTUs, we constructed partial correlation networks for rural versus urban sites and different sample types (water, surface sediment, sediment core). While partial correlation is closely related to correlation, it shows that a correlation between two variables does not necessarily mean that there is a causality relationship between them, meaning that if there is a correlation between two variables, it may be possible that this correlation can be partially explained by a third variable. In FG rural sites, strong positive co‐occurrences were mainly observed between Ruti‐Gp06, Ruti‐Ycrl, Gp05‐Myco, and Gp01‐Good (0.3 ⩽ *ρ* ⩽ 1), while the strongest negative co‐occurrences occurred between Gp03‐Ruti, Rhod‐Gp06, Gilv‐Gp06, and Ms16‐Colo (0.06 ⩽ *ρ* ⩽ 0.57) (Figure 7A). Urban sites exhibited more and stronger co‐occurrences among MOTUs, with notable positive co‐occurrences including Gp04‐Colo, Gp03‐Myco, Haem‐Rhod, Haem‐Js62, Haem‐Vanb, Gp13‐Rhod, Gp06‐Diox, and Gp08‐Gp07, whereas strong negative co‐occurrences were observed for Epa4‐Haem, Gp05‐Dj10, and Gp03 and Ms16 (Figure 7B). Co‐occurrence networks also varied by habitat type (Figure 7C–E). In water, key positive co‐occurrences included Simi‐Ruti, Smeg‐Gp13, Myco‐Aviu, Good‐Gp11, while negative co‐occurrences involved Kans‐Gp04. Surface sediments exhibited strong positive co‐occurrences between Gp08‐Gp01, Good‐Gilv, and Aviu‐Gp07, with negative co‐occurrences between Colo‐Immu, Immu‐Good, Immu‐Ms16, Gilv‐Gp08, and Gp08‐Gp09, among others. In sediment cores, positive co‐occurrences included Haem‐Pall, Ms16‐Js62, and Simi‐Gp05, while negative co‐occurrences occurred for Lito‐Ms16 and Gp13‐Shig. Overall, MOTU co‐occurrences were stronger in urban compared to rural sites and more pronounced in surface sediments than in water or sediment cores, correlating with higher MOTU richness in surface sediments. Similar trends were observed in CI, though notable differences emerged, such as Gilv‐Ruti, Gilv‐Simi, Ruti‐Simi, Ruti‐Ms16, Myco‐Epa4, and Gp09‐Gp02, Ycrl‐Smeg, Dj10‐Js62 being highly exclusive in water and sediment samples, respectively (Figure 7F,G), while strong co‐occurrences occurred between Ycrl‐Gp06 and Ms16‐Kans in water samples versus Rhod‐Ruti, Shig‐Gp01, Gp11‐Dj10, Gp06‐Js61, Epa4‐Lito in sediment samples. Overall, while strong co‐occurrences were observed for a few numbers of MOTUs, many MOTUs were widely distributed within these ecosystems without displaying co‐occurrences and located at the periphery of the network. Again, these findings suggest that mycobacterial ecological interactions may occur at the microhabitat level rather than following clear biogeographic patterns.

**FIGURE 7:**
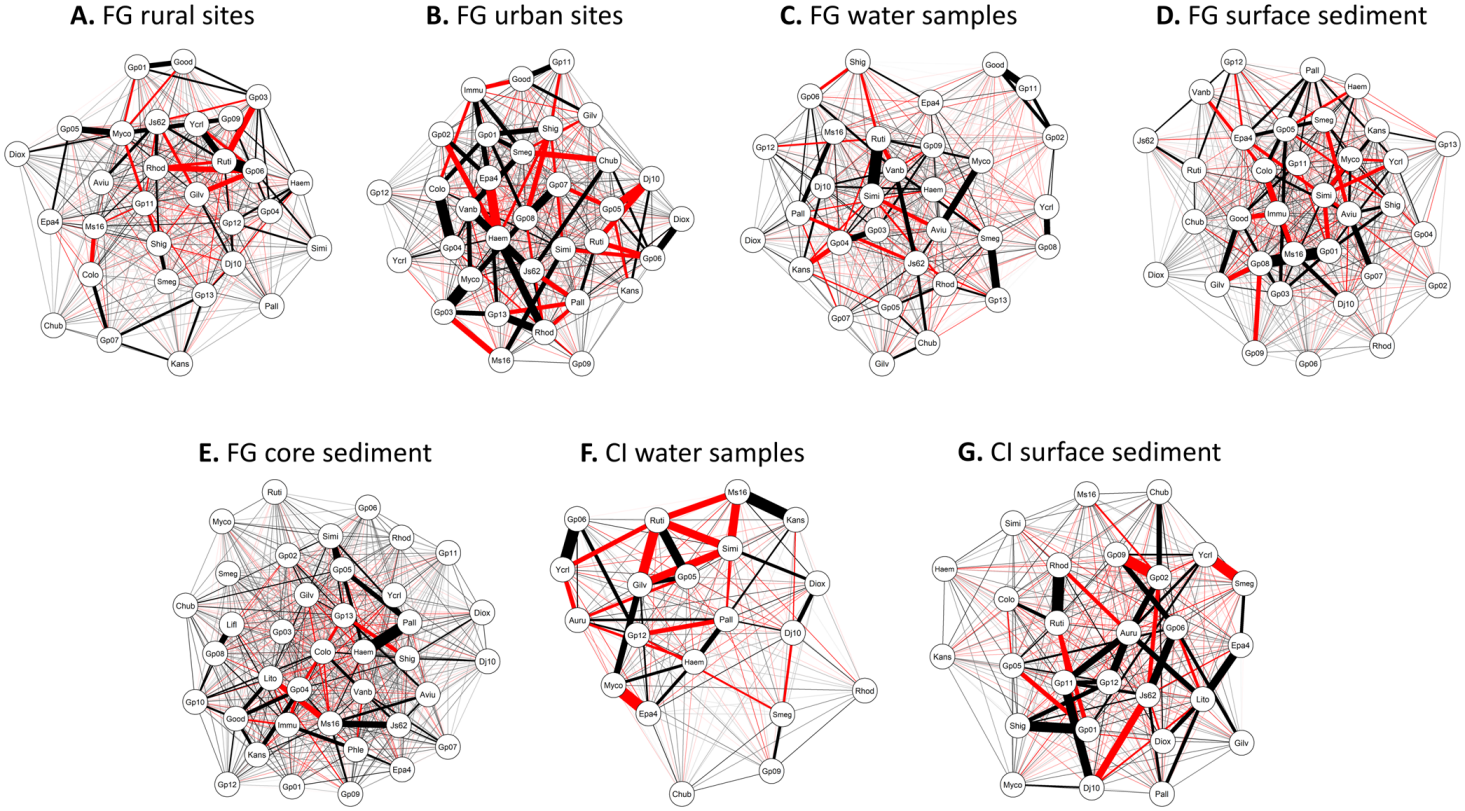
Network analysis of the partial correlation matrix between MOTUs identified in French Guiana (FG: A, B, C, D, E) and in Côte d'Ivoire (CI: F, G). The red line represents negative correlations between MOTUs and the black line positive correlations. The thicker the lines, the more important the correlation. Here, it is a network of partial correlations, the nodes represent the MOTUs and the links represent the partial correlations between these variables that is to say after control for the other variables of the network.

## 5 Discussion

Our study provides novel insights into patterns of diversity and distribution of environmental mycobacteria in freshwater habitats at a regional scale (FG) and between two countries (FG and CI). Using high‐throughput sequencing, we identified 39/42 mycobacterial species or groups of species (MOTUs), including potentially human pathogens, and demonstrated that their environmental structuration seems to be shaped by selective pressures acting at the microhabitat level. These findings have significant implications for microbial ecology, freshwater ecosystem health, and the potential emergence of zoonotic mycobacterial infections.

### 5.1 Microbial Ecology and Environmental Partitioning

In FG, mycobacterial community composition varied between urban and rural sites, between water and sediment samples, and across sediment depths. We found a greater MOTU richness in surface sediments compared to the water column, with distinct microbial assemblages between urban and rural sites, with urban sediments indeed exhibiting greater MOTU richness. These results are consistent with previous findings suggesting that sediments provide a more stable environment for microbial persistence and biofilm formation (Falkinham 2009; Tortoli 2006; Gomez‐Alvarez et al. 2021). This is also in line with previous studies showing that environmental (myco)bacteria, notably pathogenic ones (including the MPM complex) and/or those directly transmitted from the environment, exhibited higher prevalences in urban environments compared with rural ones and are constrained by local habitat characteristics such as water temperature, pH, oxygen, salinity, sedimentation and turbidity, biofilm formation, and rainfall patterns (Combe et al. 2019, 2023). Alternatively, increased nutrient availability and anthropogenic pollution in urban areas may enhance mycobacterial diversity (van Ingen et al. 2009; Combe et al. 2017).

Although our results did not show a significant difference in the pH between urban and rural sediments, the pH tended to be slightly higher in urban water compared to rural areas, and one could hypothesise that local micro‐variations in pH may influence the composition of mycobacterial communities. Our results are in line with a recent study that found that soil pH (0–10 cm) tends to be higher in urban and peri‐urban areas, which could favour the growth of certain bacterial species better adapted to these conditions (Li et al. 2023). In China, soil microbial richness was found positively correlated with pH but negatively correlated with total phosphorus (Jiao, Chen, et al. 2016; Jiao, Liu, et al. 2016). However, other studies found contrasting results depending on the studied environment; for instance, in lake sediments pH was negatively correlated with microbial richness and phylogenetic diversity (Xiong et al. 2012), while in acid soils, higher bacterial alpha diversity was exhibited (Griffiths et al. 2011). In other cases, bacterial diversity was found to be the highest for near‐neutral pH (Lauber et al. 2009). Similarly, in the present study, we did not find a difference between nitrogen levels in urban versus rural areas, potentially because in FG the anthropogenic impact and urban development are not heavy (Ortiz‐Zayas et al. 2006).

Whilst DNA sedimentation could have occurred, we believe that this effect was marginal; otherwise, we would have observed an accumulation of MOTU DNA through the sediment layers, which was not the case here. Rather, our results are in good agreement with other studies that found lower microbial abundances and biomass in deeper sol layers compared with surface layers (Fierer et al. 2003), and a decrease in the abundance and changes in the composition of microbial communities with sediment depth, notably for the *Mycobacterium* OTU (Myco) (Rissanen et al. 2019). Such observations may be explained in part by the micro‐characteristics of these ecosystems, leading to differences in the bioavailability of carbon, nitrogen, oxygen, phosphorus, pH, etc. sources necessary for these microorganisms (Gomez‐Alvarez et al. 2021).

In line with this, our results showed that phosphorus (P) and substrate type (silt vs. coarse sand) were potentially important abiotic elements in the structuring of mycobacterial communities in FG, with some mycobacteria proliferating under high levels of phosphorus (Gp13), low levels of clays, silts versus high levels of coarse sands, for instance. The positive correlation between phosphorus and *M. avium* complex (Gp13), *M. vanbaaleni* (Vanb), and *Mycobacterium* sp. *JS623* (Js62) suggests that nutrient availability may play a critical role in shaping mycobacterial diversity in tropical ecosystems such as FG, and notably in sediment layers (Jiao, Chen, et al. 2016; Jiao, Liu, et al. 2016). In line with our results, Rejas et al. (2005) showed that the reduction in bacterial abundances in depth in tropical freshwaters was mainly due to severe nutrient limitation, especially phosphorus, rather than reduced availability of organic matter. Also, the effect of soil particle size, including clay, silt, and sand, was previously proposed to contribute to spatial heterogeneity and bacterial diversity in soils, with nitrifying bacteria exhibiting highest concentrations in fine silt particles (Hemkemeyer et al. 2018). Taken together, these findings reinforce the idea that vertical stratification may play a crucial role in structuring microbial assemblages.

Interestingly, in urban and peri‐urban sites, we found shared mycobacterial MOTUs between FG and CI, suggesting that these mycobacterial communities are not specific to a region or a country. Rather, these findings support the hypothesis that local environmental factors, rather than strict biogeographical barriers, may drive mycobacterial community structure. It has been proposed that in natural soils stochasticity may remain the dominant process driving the structuration of microbial communities, but that oil contamination, for instance, could increase the impact of deterministic processes (Liang et al. 2015). Furthermore, some studies have proposed that geographic distance may limit the dispersal of microorganisms (Xiong et al. 2014; Liang et al. 2015; Wang et al. 2015; Diniz‐Filho and Telles 2000). For instance, Jiao, Chen, et al. (2016) and Jiao, Liu, et al. (2016) found that dispersal limitation played a significant impact in structuring bacterial communities in oil‐contaminated environments. Here our findings show that mycobacteria communities are indeed ubiquitous in tropical freshwater environments, but that abiotic parameters—slightly pH and more notably phosphorus levels—occurring at the micro‐habitat level (sediment layers) may trigger either a dormancy‐like state or their proliferation in the ecosystem.

### 5.2 Biotic Co‐Occurrences and Mycobacterial Network Structure

In the specific context of freshwater aquatic sites in FG, our results did not show a clear link between mycobacterial communities and those of invertebrates (Figure 6A,B). Such lack of direct correlation does not necessarily imply an absence of significant ecological interactions between both communities. Indeed, interactions between bacteria and aquatic invertebrates can be complex. Reported aquatic hosts of mycobacteria involve protozoans, including amoebae, molluscs, crustaceans, cnidarians, echinoderms, and sponges (Davidovich et al. 2020). In freshwater ecosystems, MPM have been found associated with a wide range of macro‐invertebrate taxa and exhibited higher bacterial loads found in taxa of low and intermediate trophic level (reviewed in Combe et al. 2017). Such wide host range can be explained by the widespread distribution of mycobacteria in the aquatic environment, resulting thus in the absence of correlation between mycobacterial and invertebrate communities. Alternatively, NTM have the ability to exploit ecological niches unoccupied by other (micro)organisms, a characteristic that may also partly explain why we did not observe direct biotic interactions (Davidovich et al. 2020).

Within specific ecological niches, microbial communities form complex interaction networks (Faust and Raes 2012), with some taxa being highly connected within and between ecological clusters, while other taxa will support lower number of connections within the network (Guimera and Amaral 2005; Poudel et al. 2016). Moreover, taxa highly connected within and between ecological clusters are expected to support the structure of the network (Faust et al. 2015; Toju, Tanabe, and Sato 2018) but also create ecological niche for other taxa (Toju, Peay, et al. 2018). Interaction networks are usually used to provide information into the structure and the interaction of complex microbial communities (Barberan et al. 2012) and their importance for investigating the role of microbial communities have been proven in natural ecosystems (Wagg et al. 2019; Delgado‐Baquerizo et al. 2020) and for agricultural management through manipulating microbial key taxa (Shi et al. 2020). Here, co‐occurrence network analysis revealed that in FG urban environments foster co‐occurrences between mycobacterial MOTUs compared with rural sites, suggesting that simplified urban ecosystems, with low‐level trophic networks due to biodiversity loss and human‐derived pollution (Combe et al. 2019), would favour the proliferation and the dominance of certain mycobacteria and thus enhance microbial interactions. Notable positive co‐occurrences included notably Gp04‐Colo, Gp03‐Myco, and Haem‐Rhod, while negative co‐occurrences between Epa4‐Haem and Gp05‐Dj10 potentially highlighted competitive dynamics driven by niche differentiation and resource availability. Urban environments are well‐known to be more suitable to pathogens. For instance, urban sewage water and standing water are known to represent a major reservoir for pathogenic *Leptospira* sp. (Casanovas‐Massana et al. 2018; Combe et al. 2019); water‐borne and enteric viral and bacterial diseases are at high risk in urban areas, notably those with poor sanitation infrastructure (Cable et al. 2017). Co‐occurrences also varied by habitat type in FG and in CI, with water samples showing fewer significant co‐occurrences than surface sediments. Again, this aligns with the notion that urban sediment matrices provide a more structured environment conducive to microbial consortia formation (Doloman and Sousa 2024; Gomez‐Alvarez et al. 2021).

We also found that only a few numbers of MOTUs were frequently involved in such co‐occurences, potentially suggesting their importance in the structuring and the functioning of microbial networks and more widely of the ecosystem. It is for instance the case for *M. rutilum* (Ruti), *M. haemophilum* (Haem), *M. immunogenum* (Immu), *M. gilvum* (Gilv) and *M. simiae* (Simi). *M. rutilum* and *M. gilvum* , that also have the ability to degrade pollutants such as polycyclic aromatic hydrocarbons (PAH), including phenanthrene (PHE) and pyrene (PYR), which are highly toxic, carcinogenic and mutagenic compounds widely distributed in the environment and more frequently encountered in urban soils (Rein et al. 2016). Based on these findings, one could hypothesise that these mycobacteria may function as environmental purifiers, also potentially explaining their strong co‐occurrence as key species within mycobacterial networks in FG and CI. However, only a deeper analysis of the functional roles of these specific mycobacteria would help to resolve this hypothesis.

When focusing on specific mycobacteria, *M. haemophilum* is an emerging pathogen in humans with a reported unknown natural reservoir. Our study provides thus novel information about the natural environment of this mycobacterium, since we found its DNA in both countries (FG and CI), but mainly showing potential strong ecological interactions in urban sites in FG. Similarly, our study revealed the natural reservoir of *M. simiae* , which remained uncertain in the literature. *M. immunogenum* is reported to be resistant to disinfection procedures, including water chlorination, ozone and addition to biocides, conditions which in fact select for more resistant mycobacterium and provide thus a preferential ecological niche (Primm et al. 2004).

Microbial communities are well known to represent a critical component to the functioning of the ecosystem, playing key roles in nutrient cycling but also food safety and disease emergence (Lin et al. 2023). Microbial communities are also known to interact within ecological niches and it has been suggested that these ‘micro‐communities’ may function collectively (Hirano and Takemoto 2019), and exhibit social behaviours such as biofilm formation and quorum sensing as a result of inter‐ and intra‐species interactions ranging from competition for nutrients to cooperative networks (i.e., metabolite exchanges) (Antoniewicz 2020). Whilst complex interactions within microbial communities often prevent their exact characterisation, a better understanding of the nature of these interactions can boost our understanding of their resilience and function (Lin et al. 2023). For instance, it has been proposed that spatially structured microbial communities tend to favour cooperative behaviours (e.g., quorum sensing, commensalism). Contrarily, spatially unstructured or well‐mixed communities mainly exhibit competition (e.g., parasitism, amensalism) (Nadell et al. 2016). Whilst our data could not assess the mechanisms involved in mycobacterial interactions between mycobacterial MOTU, with a trend towards potential exclusions. Overall, our results tend to suggest that mycobacterial communities are extremely diverse in freshwater environments in FG and in CI; while some exhibit a distribution pattern specific to the microhabitat, with a few dominant MOTU being specific to certain habitat types versus a large number of low‐abundance and/or low‐prevalence MOTU being more ubiquitous in these environments, potentially corresponding to the ‘rare biosphere’ (Nemergut et al. 2013). In other words, we propose that whilst most MOTUs should be find everywhere in freshwater tropical ecosystems, certain mycobacteria may have preferential niches within aquatic environments, likely influenced by competition, predation, and symbiotic relationships.

### 5.3 Implications for Infectious Disease Emergence

Importantly, our results revealed the potential (either as unique species or as a group of species) widespread presence of several pathogenic mycobacteria in tropical water and sediment samples, including the *M. avium* complex (Gp13), in freshwater environments, raising concerns about potential human and animal exposure risks. Water is widely recognised to represent the primary source of *M. avium* complex (Gp13) infection in humans, and DNA‐based fingerprints of *M. avium* isolates from AIDS patients were identical to the isolates found in the patient's drinking water (Primm et al. 2004). In FG and CI, we found *M. haemophilum* , a bacterium causing fish mycobacteriosis (Yanong et al. 2010) but also reported as an emerging mycobacterium causing localised skin infections or even a disseminated disease such as septic arthritis, osteomyelitis, and pneumonitis in immunocompromised hosts, notably in children (Lindeboom et al. 2011). Interestingly, while not belonging to the MPM complex usually known to cause Buruli ulcer, the disease caused by *M. haemophilum* most resembles those of *M. marinum* and *M. ulcerans* , as is the case for its genomic traits compared with other mycobacteria (Lindeboom et al. 2011). Also, some similarities, in terms of fatty acids content and proteic and lipidic properties, have been identified between this mycobacterium and *M. leprae* (Besra et al. 1991). Similarly, *M. simiae* is known to cause mycobacteriosis in fish (Toranzo 2004) and cervical lymphadenitis in humans (Cruz and Palazzi 2007). *M. immunogenum* , only found in FG in this study, is known to cause hypersensitive pneumonitis in humans (Selman et al. 2012).

*M. gilvum* (Gilv) is a fast‐growing microorganism that was previously found in sputum samples (Stanford and Gunthorpe 1971), pulmonary tuberculosis infections in Brazil (Mendes de Lima et al. 2013), and in bathing water of leprosy patients in West Bengal (Lavania et al. 2014). Also, *M. gilvum* has been isolated from river sediments based on its ability to degrade polycyclic aromatic hydrocarbons (PAH), such as pyrene, as growth substrate (Lamrabet and Drancourt 2013). Whilst this mycobacterium was believed to be non‐pathogenic, it has been proposed to act as an immunomodulator of the host immune system towards susceptibility to other mycobacteria such as *M. leprae* (Lavania et al. 2014). Moreover, *M. gilvum* has been found as a single mycobacterium or as co‐infections in Buruli ulcer skin biopsies in FG, raising questions about its mechanism of pathogenicity (Combe et al. 2020). Here, the widespread presence of *M. gilvum* across all habitat types (urban, rural, water, sediments) and its high prevalence in FG and in CI (i) suggest that certain environmental mycobacteria may serve as indicators of ecosystem health and anthropogenic impact and (ii) raise concerns about opportunistic infections. Overall, given the increasing incidence of NTM infections, particularly in immunocompromised populations, our findings emphasise the importance of monitoring mycobacterial communities in freshwater environments as potential sources of opportunistic infections (Johansen et al. 2020).

Surprisingly, our eDNA‐based metabarcoding screening detected the Mycolactone‐Producing‐Mycobacteria (MPM) complex, which includes closely related variants such as *M. ulcerans* , *M. shinshuense* , *M. pseudoshottsii* , *M. liflandii* , and *M. marinum* (Combe et al. 2023), at very low environmental prevalence and only in FG (MOTUs Lifl and Gp08). However, all these variants have the ability to cause Buruli ulcer and were previously found in patient biopsies in Japan, CI, and FG (reviewed in Combe et al. 2023). Whilst Buruli ulcer remains a neglected disease, its importance is no less since it affects nearly 5000–6000 people annually across 33 countries and represents the third most common mycobacterial disease after tuberculosis and leprosy (reviewed in Combe et al. 2023). Consequently, the observed very low prevalence of these mycobacteria in freshwater matrices in FG and their absence from CI suggests that the transmission dynamics of the causative mycobacterial agents causing Buruli ulcer may involve complex ecological reservoirs or undetected amplification hosts. This finding is particularly relevant given the endemicity of Buruli ulcer in West Africa (Tabah et al. 2019). In addition, our results suggest that although eDNA‐based metabarcoding approaches are very successful in assessing microbial species diversity in space and time, cryptic microbial species may remain undetected. By contrast, qPCR assays targeting specific species—which are known to be more sensitive and specific methods—seem more efficient in identifying those cryptic pathogens. These discrepancies also underscore the need for multi‐marker and/or approaches to improve pathogen surveillance in aquatic ecosystems (Mve‐Obiang et al. 2003).

Overall, the greater MOTU richness found in urban sediment samples, and the discrepancy between microbial assemblages between urban and rural areas, may have important implications for public health and urbanisation. For instance, it is well known that soil represents a reservoir for the *M. avium* complex (Gp13) associated with human infection, and that individuals whose occupations involve prolonged exposure to soil are at increased risk of infection (Reed et al. 2006). Similarly, our current understanding of Buruli ulcer disease relies on higher prevalence of the MPM variants in urban environments and notably in sediment samples (Combe et al. 2019). In fact, freshwater habitats represent the natural reservoir of the mycobacterium responsible for Buruli ulcer, which are spread into new habitats during flooding. However, human‐disturbed environments, following for instance deforestation, agriculture and urbanisation and leading to stagnant water bodies with increased water temperature, decreased pH and oxygen levels, algae growth, biofilm formation and also exhibiting sudden changes in food web structure and function, are at higher risk of disease emergence (reviewed in Combe et al. 2017). Similarly, other mycobacterium, such as *M. avium* (Aviu) and *M. intracellulare* (Gp10), exhibit an acidic pH optimum comprised between 4.5–5.5 (George and Falkinham III 1986), and their growth is enhanced by humic and fulvic acids, the major compounds found in brown water swamps (Kirschner et al. 1999). Higher bacterial loads of *M. avium* and *M. intracellulare* have been found in water and soils of low oxygen levels (Brooks et al. 1984; Kirschner et al. 1992). Together, these findings strengthen the fact that such environmental conditions, prone to increased virulence and pathogenicity (Primm et al. 2004), are more frequently encountered in urban areas where animal and human exposure is also enhanced.

In urban areas human‐mediated pollution may explain the occurrence of many mycobacterial species known for their ability to degrade pollutants such as PAH, such as *M. rutilum* (Ruti), *M. gilvum* (Gilv), *M. haemophilum* (Haem) or *M. immunogenum* (Immu), among others. The presence of a greater richness of mycobacterial species in urban areas, together with pollution and the increased release of antimicrobial drugs (antibiotics) and disinfectant (chlorine, ozone, biocides) compared with rural areas, represent selective environments could boost either the acquisition or the expression of resistance genes by these opportunistic mycobacteria (Norton and LeChevallier 2000). Indeed, the prevalence of environmental mycobacteria in municipal drinking water supplies is directly explained by their high innate chlorine, ozone and biocide resistance (Le Dantec et al. 2002; Norton and LeChevallier 2000; Taylor et al. 2000). For comparison, *M. aurum* mycobacterium is related to be 100‐fold more resistant to chlorine than *Escherichia coli* (Le Dantec et al. 2002).

Interestingly, our results suggest that although eDNA‐based metabarcoding approaches are very successful in assessing microbial species diversity in space and time, cryptic microbial species may remain undetected. By contrast, qPCR assays targeting specific species—which are known to be more sensitive and specific methods—seem more efficient in identifying those cryptic pathogens. These discrepancies also underscore the need for multi‐marker and/or approaches to improve pathogen surveillance in aquatic ecosystems (Mve‐Obiang et al. 2003). Overall, our study provides new insight into patterns of the diversity and distribution of environmental mycobacteria in tropical freshwater ecosystems. We suggest that a deeper understanding of mycobacterial distribution patterns in the environment could help to identify hotspots of infectious disease risk in order to guide targeted surveillance programmes. Future studies conducted in other tropical countries should thus confirm the patterns observed here and perform functional analysis of each MOTU identified in order to reveal ecological roles and potential interactions between NTM.

## 6 Conclusions

This study advances our understanding of environmental mycobacteria ecology by demonstrating the influence of habitat type, abiotic factors, and potential microbial ecological interactions on mycobacterial distribution. The potential detection of several pathogenic species from freshwater environments suggests that under specific environmental conditions these pathogens could emerge, further highlighting the importance of environmental reservoirs in mycobacterial disease emergence and transmission. Future research should focus on integrating metagenomics and functional analyses to elucidate the ecological roles of mycobacteria in freshwater ecosystems and their potential public health implications. Longitudinal studies incorporating climate variability and land‐use changes may also provide further insights into the environmental drivers of mycobacterial diversity and their relevance to zoonotic disease emergence.

## Author Contributions

**Marine Combe:** conceptualization, investigation, writing – original draft, methodology, validation, visualization, supervision. **Amar Bouam:** investigation, writing – review and editing. **Sylvestre Dizoe:** investigation. **Bernard Davoust:** investigation. **Emilien Drancourt:** investigation. **Dylan Messeca:** software, formal analysis, data curation. **Alice Valentini:** methodology, validation, writing – review and editing, software, formal analysis, data curation. **Romain Blaizot:** writing – review and editing. **Rodolphe Elie Gozlan:** conceptualization, investigation, funding acquisition, writing – review and editing, supervision.

## Funding

This work was supported by Centre d'Étude de la Biodiversité Amazonienne (ANR‐10‐LABX‐25‐01) and Agence Nationale de la Recherche (ANR‐17‐CE35‐0006‐01).

## Conflicts of Interest

A.V. is a research scientist in a private company specialising in the use of eDNA for biodiversity monitoring, with some patented technologies (SPYGEN). The other authors declare no conflicts of interest.

## Supporting information


**Figure S1:** Some aquatic sites sampled in French Guiana (FG). (A) Amec; (B) Remi; (C) Cri2; (D) Cay3. See Table 1 for site names.
**Figure S2:** Some aquatic sites sampled in Côte d'Ivoire (CI). (A) ALO01; (B) BO01; (C) KO01; (D) GEO3. See Table 1 for site names.
**Figure S3:** Reads generated by Illumina MiSeq. The total number of reads is represented by the black lines whilst the red lines show the number of reads assigned to a NTM MOTU after applying the 97% similarity treshold. (A) reads in water and surface sediment samples in French Guiana; (B) reads in sediment cores in French Guiana; (C) reads in water and surface sediment in Côte d'Ivoire.
**Figure S4:** Ven diagram of the MOTU found in FG and CI.
**Figure S5:** Score (area under ROC curve) of the contributions of each variable (mycobacterial MOTUs) in the LDA analysis. The higher the score for a MOTU, the more important it is for distinguishing between the two groups of sites observed (rural and urban sites).
**Figure S6:** Multidimensional Non‐Metric Scales (NMDS) analysis on a dissimilarity matrix (Jaccard) and NMDS Stressplot for urban sites. The name of each site is indicated, and the letter S denotes the MOTU diversity of mycobacteria based on a surface sediment sample, and the letter W (water) for a water sample. Sites further apart on the NMDS axes indicate that they share few species in common. A stress < 0.05 indicates excellent representation; a stress < 0.1 is considered very good; a stress < 0.2 is acceptable; a stress > 0.3 indicates poor representation.
**Figure S7:** Distribution of the number of MOTUs per core layer for the 4 sites combined (Amec, Remi, Cay3, Cri2). The Cay3_5–10 cm value was not representative and was thus excluded from the analysis. The anova test performed shows no significative differences in the number of MOTU between sediment layers. The black bar indicates the median and the whiskers the extremum. The circle indicates an extreme value, i.e., greater than 1.5 times the interquartile range.
**Figure S8:** Distribution of the number of MOTUs per site. The Cay3_5–10 cm value was excluded as it was not representative. For the 4 sites, all sediment layers (0–1 cm, 1–2 cm, 2–3 cm, 3–4 cm, 4–5 cm, 5–10 cm) were combined. The Kruskal Wallis test shows differences between the site Amec and the sites Cay3 and Cri2. The black bar indicates the median and the whiskers the extremum. The circle indicates an extreme value i.e., greater than 1.5 times the interquartile range and the segments with ‘*’ represent significant contrasts.
**Figure S9:** Hierarchical cluster analysis (HCA) on log10(*x* + 1) transformed core data. The Cay3_5–10 cm value was removed since it was not representative. The distance used is Euclidean and the wardD2 jump was chosen. Site names (Amec, Remi, Cri2, Cay3, see Table 1) and sediment layers (1–2 cm, 2–3 cm, 3–4 cm, 4–5 cm, 5–10 cm) are indicated.
**Figure S10:** Score (Areas under ROC curves) of the importance of the LDA variables. The Cay3_5–10 cm value, not being representative, was excluded. The higher the score for a variable, the more important it is for the distinction between the two groups of observed sites (Ame/Rem and Cay3/Cri2).
**Figure S11:** Distribution of abiotic factors according to the type of site (U: urban or R: rural) collected from the water column in French Guiana (FG). (A) Temperature (°C); (B) Conductivity; (C) Oxygen; (D) pH. The black bar indicates the median and the whiskers the extremum. The circle indicates an extreme value, i.e., greater than 1.5 times the interquartile range.
**Figure S12:** Substrate distribution (%) for each site and for the sediment layers 0–5 cm and 5–10 cm. Cl.: Clay; C.Slt.: Coarse silts; C.Sd.: Coarse sands; F.Slt.: Fine silts and F.Sd.: Fine sands.
**Figure S13:** Score (Air under ROC curve) of the contributions of each MOTU (panel A) and each substrate (panel B) in the RF analyses. The higher the score, the more important it is for the distinction between the two groups of observed sites (Amec/Remi and Cay3/Cri2).
**Figure S14:** Distribution of the MOTU Haem (panel A) and Pall (panel B) according to the 4 sites analysed for sedimentary layers (0–5 cm, 5–10 cm). The black bar indicates the median and the whiskers indicate the extremum.
**Figure S15:** Distribution of substrate variables (Cl.: Clay; F.Slt.: Fine silts; C.Slt: Coarse silts; F.Sd.: Fine sands; C.Sd.: Coarse sands) according to the 4 sites analyse for sediment layers (0–5 cm, 5–10 cm). The black bar indicates the median and the whiskers indicate the extremum.
**Figure S16:** Score (air under ROC curve) of the contributions of each MOTU (Panel A) and each substrate (Panel B) in the RF analyses. The higher the score, the more important it is for the distinction between sediment layers (0–5 cm and 5–10 cm). The Cay3_5–10 cm value was excluded as it was not representative.
**Figure S17:** Distribution of F.Sd. according to sediment layers (0–5 cm, 5–10 cm). The black bar indicates the median and the whiskers indicate the extremum.
**Figure S18:** Distribution of gp1 (A), gil (B) and dio (C) according to sedimentary layers (0–5 cm, 5–10 cm). The black bar indicates the median and the whiskers represent the extremum.
**Figure S19:** Accumulation curve of the number of MOTUs in French Guiana (green) and Côte d'Ivoire (pink) across all sampled sites (12 sites for each country).
**Figure S20:** MOTU prevalence (%) in FG (A) and CI (B).
**Figure S21:** Score (Area under ROC curves) of the importance of the RF variables of the FG and CI data. The higher the score for a MOTU, the more important it is for the distinction between the two groups of sites of the two countries observed (rural and urban).
**Table S1:** Abiotic parameters of the study sites in French Guiana (FG). W: Water; Temp: Temperature (°C); Cond: Conductivity; O_2_: Oxygen; P%: Phosphorus (%); C%: Carbon (%); N%: Nitrogen (%); C/N: ration carbon/nitrogen; Cl.%: Clay (%); F.Slt.%: fine silt (%); C.Slt.%: coarse silt (%); F.Sd.%: fine sand (%); C.Sd.%: coarse sand (%). NA: unknown values.
**Table S2:** Invertebrate taxa collected in French Guiana (FG) and used to perform the analysis.
**Table S3:** Mycobacterial DNA used as positive control for the 16S rDNA PCR amplification and Illumina sequencing.
**Table S4:** List of mycobacterial species used in this study and accession Numbers where the 16S sequences were recovered.
**Table S5:** Known pathogenic environmental mycobacteria with confirmed or suspected waterborne transmission. MAC; *Mycobacterium avium* Complex which include *M. intracellulare* , *M. avium* and its subspecies *avium* (MAA), *paratuberculosis* (MAP) and *sylvaticum*. Non‐exhaustive list.

## Data Availability

The data that support the findings of this study (including raw reads) are available in the Supporting Information of this article. Raw reads are available upon request.

## References

[emi70465-bib-0001] Amha, Y. M. , M. Z. Anwar , R. Kumaraswamy , A. Henschel , and F. Ahmad . 2017. “Mycobacteria in Municipal Wastewater Treatment and Reuse: Microbial Diversity for Screening the Occurrence of Clinically and Environmentally Relevant Species in Arid Regions.” Environmental Science & Technology 51, no. 5: 3048–3056. 10.1021/acs.est.6b05580. 28139909

[emi70465-bib-0002] Antoniewicz, M. R. 2020. “A Guide to Deciphering Microbial Interactions and Metabolic Fluxes in Microbiome Communities.” Current Opinion in Biotechnology 64: 230–237. 10.1016/j.copbio.2020.07.001. 32711357

[emi70465-bib-0003] Barberan, A. , S. T. Bates , E. O. Casamayor , and N. Fierer . 2012. “Using Network Analysis to Explore Co‐Occurrence Patterns in Soil Microbial Communities.” ISME Journal 6: 343. 10.1038/ismej.2011.119. PMC3260507 21900968

[emi70465-bib-0004] Bass, D. , G. D. Stentiford , H.‐C. Wang , B. Koskella , and C. R. Tyler . 2019. “The Pathobiome in Animal and Plant Diseases.” Trends in Ecology & Evolution 34, no. 11: 996–1008. 10.1016/j.tree.2019.07.012. PMC7479508 31522755

[emi70465-bib-0005] Becking, L. B. 1934. Geobiologie of Inleiding Tot de Milieukunde (No. 18‐19). WP Van Stockum & Zoon.

[emi70465-bib-0006] Bellemain, E. , T. Carlsen , C. Brochmann , E. Coissac , P. Taberlet , and H. Kauserud . 2010. “ITS as an Environmental DNA Barcode for Fungi: An In Silico Approach Reveals Potential PCR Biases.” BMC Microbiology 10, no. 1: 1–9. 10.1186/1471-2180-10-189. PMC2909996 20618939

[emi70465-bib-0007] Besra, G. S. , M. McNeil , D. E. Minnikin , F. Portaels , M. Ridell , and P. J. Brennan . 1991. “Structural Elucidation and Antigenicity of a Novel Phenolic Glycolipid Antigen From *Mycobacterium haemophilum* .” Biochemistry 30, no. 31: 7772–7777. 10.1021/bi00245a015. 1868054

[emi70465-bib-0008] Boyer, F. , C. Mercier , A. Bonin , Y. Le Bras , P. Taberlet , and E. Coissac . 2016. “ obitools: A unix‐Inspired Software Package for dna Metabarcoding.” Molecular Ecology Resources 16, no. 1: 176–182. 10.1111/1755-0998.12428. 25959493

[emi70465-bib-0009] Brooks, R. W. , K. L. George , B. C. Parker , J. O. Falkinham , and G. Gruff . 1984. “Recovery and Survival of Nontuberculous Mycobacteria Under Various Growth and Decontamination Conditions.” Canadian Journal of Microbiology 30: 1112–1117. 10.1139/m84-174. 6509392

[emi70465-bib-0010] Cable, J. , I. Barber , B. Boag , et al. 2017. “Global Change, Parasite Transmission and Disease Control: Lessons From Ecology.” Philosophical Transactions of the Royal Society, B: Biological Sciences 372, no. 1719: 20160088. 10.1098/rstb.2016.0088. PMC5352815 28289256

[emi70465-bib-0011] Cangelosi, G. , J. Clark‐Curtis , M. Behr , T. Bull , and T. Stinear . 2004. “Biology of Waterborne Pathogenic Mycobacteria.” In Pathogenic Mycobacteria in Water. A Guide to Public Health Consequences, Monitoring and Management. In Emerging Issues in Water and Infectious Disease Series, edited by S. Pedley , J. Bartram , G. Rees , et al. World Health Organization by IWA Publishing.

[emi70465-bib-0012] Cantera, I. , K. Cilleros , A. Valentini , et al. 2019. “Optimizing Environmental DNA Sampling Effort for Fish Inventories in Tropical Streams and Rivers.” Scientific Reports 9: 3085. 10.1038/s41598-019-39399-5. PMC6395586 30816174

[emi70465-bib-0013] Casanovas‐Massana, A. , F. Costa , I. N. Riediger , et al. 2018. “Spatial and Temporal Dynamics of Pathogenic Leptospira in Surface Waters From the Urban Slum Environment.” Water Research 130: 176–184. 10.1016/j.watres.2017.11.068. PMC5767135 29220718

[emi70465-bib-0014] Clare, E. L. , C. K. Economou , C. G. Faulkes , et al. 2021. “eDNAir: Proof of Concept That Animal DNA Can Be Collected From Air Sampling.” PeerJ 9: e11030. 10.7717/peerj.11030. PMC8019316 33850648

[emi70465-bib-0015] Combe, M. , E. Cherif , R. Blaizot , D. Breugnot , and R. E. Gozlan . 2023. “What About Current Diversity of Mycolactone‐Producing Mycobacteria? Implication for the Diagnosis and Treatment of Buruli Ulcer.” International Journal of Molecular Sciences 24, no. 18: 13727. 10.3390/ijms241813727. PMC10531242 37762030

[emi70465-bib-0016] Combe, M. , P. Couppié , R. Blaizot , A. Valentini , and R. E. Gozlan . 2020. “Are All Buruli Ulcers Caused by *Mycobacterium ulcerans* ?” British Journal of Dermatology 183, no. 5: 968–970. 10.1111/bjd.19260. 32470150

[emi70465-bib-0017] Combe, M. , and R. E. Gozlan . 2024. “When the Blue Marble Health Concept Challenges Our Current Understanding of One Health.” One Health 19: 100935. 10.1016/j.onehlt.2024.100935. PMC11617403 39640907

[emi70465-bib-0018] Combe, M. , R. E. Gozlan , S. Jagadesh , et al. 2019. “Comparison of *Mycobacterium ulcerans* (Buruli Ulcer) and *Leptospira* sp. (Leptospirosis) Dynamics in Urban and Rural Settings.” PLoS Neglected Tropical Diseases 13, no. 1: e0007074. 10.1371/journal.pntd.0007074. PMC6336349 30615683

[emi70465-bib-0019] Combe, M. , C. J. Velvin , A. Morris , et al. 2017. “Global and Local Environmental Changes as Drivers of Buruli Ulcer Emergence.” Emerging Microbes & Infections 6: e20. 10.1038/emi.2017.7. PMC5520486 28487559

[emi70465-bib-0020] Crooks, G. E. , G. Hon , J. M. Chandonia , and S. E. Brenner . 2004. “WebLogo: A Sequence Logo Generator.” Genome Research 14, no. 6: 1188–1190. 10.1101/gr.849004. PMC419797 15173120

[emi70465-bib-0021] Cruz, A. T. , and D. L. Palazzi . 2007. “An Adolescent With Fever, Cough, & Diffuse Lymphadenopathy.” Pediatric Infectious Disease Journal 26, no. 3: 275. 10.1097/01.inf.0000256756.70575.4d. 17484234

[emi70465-bib-0022] Curtis, T. P. , W. T. Sloan , and J. W. Scannell . 2002. “Estimating Prokaryotic Diversity and Its Limits.” Proceedings of the National Academy of Sciences 99, no. 16: 10494–10499. 10.1073/pnas.142680199. PMC124953 12097644

[emi70465-bib-0023] Daszak, P. , A. A. Cunningham , and A. D. Hyatt . 2000. “Emerging Infectious Diseases of Wildlife – Threats to Biodiversity and Human Health.” Science 287, no. 5452: 443–449. 10.1126/science.287.5452.44. 10642539

[emi70465-bib-0024] Davidovich, N. , D. Morick , and F. Carella . 2020. “Mycobacteriosis in Aquatic Invertebrates: A Review of Its Emergence.” Microorganisms 8, no. 8: 1249. 10.3390/microorganisms8081249. PMC7464023 32824567

[emi70465-bib-0025] De Groote, M. A. , and P. Johnson . 2004. “Pathogenic Mycobacteria in Water. A Guide to Public Health Consequences, Monitoring and Management.” In Emerging Issues in Water and Infectious Disease Series, edited by S. Pedley , J. Bartram , G. Rees , et al. World Health Organization by IWA Publishing.

[emi70465-bib-0026] Delgado‐Baquerizo, M. , P. B. Reich , C. Trivedi , et al. 2020. “Multiple Elements of Soil Biodiversity Drive Eco‐ System Functions Across Biomes.” Nature Ecology & Evolution 4: 210–220. 10.1038/s41559-019-1084-y. 32015427

[emi70465-bib-0027] Diniz‐Filho, J. A. F. , and M. P. C. Telles . 2000. “Spatial Pattern and Genetic Diversity Estimates Are Linked in Stochastic Models of Population Differentiation.” Genetics and Molecular Biology 23: 541. 10.1590/S1415-47572000000300007.

[emi70465-bib-0028] Doloman, A. , and D. Z. Sousa . 2024. “Mechanisms of Microbial Co‐Aggregation in Mixed Anaerobic Cultures.” Applied Microbiology and Biotechnology 108, no. 1: 407. 10.1007/s00253-024-13246-8. PMC11224092 38963458

[emi70465-bib-0029] Douine, M. , R. E. Gozlan , M. Nacher , et al. 2017. “ *Mycobacterium ulcerans* Infection (Buruli Ulcer) in French Guiana, South America, 1969‐2013: An Epidemiologic Study.” Lancet Planetary Health 1: e65–e73. 10.1016/S2542-5196(17)30009-8. 29851583

[emi70465-bib-0030] Drummond, A. J. , R. D. Newcomb , T. R. Buckley , et al. 2015. “Evaluating a Multigene Environmental DNA Approach for Biodiversity Assessment.” GigaScience 4, no. 1: 46. 10.1186/s13742-015-0086-1. PMC4595072 26445670

[emi70465-bib-0031] Elliott, I. , M. Combe , A. Valentini , and R. E. Gozlan . 2026. “Environmental DNA or Traditional Sampling Approaches: The Best of Both Worlds for Assessing Tropical Freshwater Biodiversity.” Water Biology and Security 100629: 100629. 10.1016/j.watbs.2026.100629.

[emi70465-bib-0032] Epskamp, S. , A. O. Cramer , L. J. Waldorp , V. D. Schmittmann , and D. Borsboom . 2012. “Qgraph: Network Visualizations of Relationships in Psychometric Data.” Journal of Statistical Software 48: 1–18. 10.18637/jss.v048.i04.

[emi70465-bib-0033] Falkinham, J. O. 2002. “Nontuberculous Mycobacteria in the Environment.” Clinics in Chest Medicine 23, no. 3: 529–551. 10.1016/S0272-5231(02)00014-X. 12370991

[emi70465-bib-0034] Falkinham, J. O. 2009. “Surrounded by Mycobacteria: Nontuberculous Mycobacteria in the Human Environment.” Journal of Applied Microbiology 107, no. 2: 356–367. 10.1111/j.1365-2672.2009.04161.x. 19228258

[emi70465-bib-0035] Faust, K. , G. Lima‐Mendez , J. S. Lerat , et al. 2015. “Cross‐Biome Comparison of Microbial Association Networks.” Frontiers in Microbiology 6: 1200. 10.3389/fmicb.2015.01200. PMC4621437 26579106

[emi70465-bib-0036] Faust, K. , and J. Raes . 2012. “Microbial Interactions: From Networks to Models.” Nature Reviews Microbiology 10: 538. 10.1038/nrmicro2832. 22796884

[emi70465-bib-0037] Ficetola, G. F. , E. Coissac , S. Zundel , et al. 2010. “An In Silico Approach for the Evaluation of DNA Barcodes.” BMC Genomics 11, no. 1: 1–10. 10.1186/1471-2164-11-434. PMC3091633 20637073

[emi70465-bib-0038] Fierer, N. , and R. B. Jackson . 2006. “The Diversity and Biogeography of Soil Bacterial Communities.” Proceedings of the National Academy of Sciences of the United States of America 103, no. 3: 626–631. 10.1073/pnas.050753510. PMC1334650 16407148

[emi70465-bib-0039] Fierer, N. , J. P. Schimel , and P. A. Holden . 2003. “Variations in Microbial Community Composition Through Two Soil Depth Profiles.” Soil Biology and Biochemistry 35, no. 1: 167–176. 10.1016/S0038-0717(02)00251-1.

[emi70465-bib-0040] George, K. L. , and J. O. Falkinham III . 1986. “Selective Medium for the Isolation and Enumeration of *Mycobacterium avium* ‐Intracellulare and *M. scrofulaceum* .” Canadian Journal of Microbiology 32, no. 1: 10–14. 10.1139/m86-003. 3697842

[emi70465-bib-0041] Gomez‐Alvarez, V. , H. Liu , J. G. Pressman , and D. G. Wahman . 2021. “Metagenomic Profile of Microbial Communities in a Drinking Water Storage Tank Sediment After Sequential Exposure to Monochloramine, Free Chlorine, and Monochloramine.” ACS ES&T Water 1, no. 5: 1283–1294. 10.1021/acsestwater.1c00016. PMC8318090 34337601

[emi70465-bib-0042] Griffiths, R. I. , B. C. Thomson , P. James , T. Bell , M. Bailey , and A. S. Whiteley . 2011. “The Bacterial Biogeography of British Soils.” Environmental Microbiology 13: 1642. 10.1111/j.1462-2920.2011.02480.x. 21507180

[emi70465-bib-0043] Guimera, R. , and L. A. N. Amaral . 2005. “Functional Cartography of Complex Metabolic Networks.” Nature 433: 895–900. 10.1038/nature03288. PMC2175124 15729348

[emi70465-bib-0044] Hammoudi, N. , S. Dizoe , J. Saad , et al. 2020. “Tracing *Mycobacterium ulcerans* Along an Alimentary Chain in Côte d'Ivoire: A One Health Perspective.” PLoS Neglected Tropical Diseases 14, no. 5: e0008228. 10.1371/journal.pntd.0008228. PMC7255608 32463813

[emi70465-bib-0045] Hemkemeyer, M. , A. B. Dohrmann , B. T. Christensen , and C. C. Tebbe . 2018. “Bacterial Preferences for Specific Soil Particle Size Fractions Revealed by Community Analyses.” Frontiers in Microbiology 9: 149. 10.3389/fmicb.2018.00149. PMC5829042 29527192

[emi70465-bib-0046] Hirano, H. , and K. Takemoto . 2019. “Difficulty in Inferring Microbial Community Structure Based on Co‐Occurrence Network Approaches.” BMC Bioinformatics 20: 329. 10.1186/s12859-019-2915-1. PMC6567618 31195956

[emi70465-bib-0047] Horner‐Devine, M. C. , M. Lage , J. B. Hughes , and J. M. Bohannan . 2004. “A Taxa‐Area Relationship for Bacteria.” Nature 432: 750–753. 10.1038/nature03073. 15592412

[emi70465-bib-0048] Jagadesh, S. , M. Combe , P. Couppié , et al. 2019. “Emerging Human Infectious Diseases of Aquatic Origin: A Comparative Biogeographic Approach Using Bayesian Spatial Modelling.” International Journal of Health Geographics 18, no. 1: 23. 10.1186/s12942-019-0188-6. PMC6833193 31694656

[emi70465-bib-0049] Jagadesh, S. , M. Combe , P. Couppié , M. Nacher , and R. E. Gozlan . 2019. “Global Emergence of Buruli Ulcer.” EcoHealth 16: 591–593. 10.1007/s10393-019-01445-z. 31628633

[emi70465-bib-0050] Jiao, S. , W. Chen , E. Wang , et al. 2016. “Microbial Succession in Response to Pollutants in Batch‐Enrichment Culture.” Scientific Reports 6, no. 1: 21791. 10.1038/srep21791. PMC4764846 26905741

[emi70465-bib-0051] Jiao, S. , Z. Liu , Y. Lin , J. Yang , W. Chen , and G. Wei . 2016. “Bacterial Communities in Oil Contaminated Soils: Biogeography and Co‐Occurrence Patterns.” Soil Biology and Biochemistry 98: 64–73. 10.1016/j.soilbio.2016.04.005.

[emi70465-bib-0052] Johansen, M. D. , J. L. Herrmann , and L. Kremer . 2020. “Non‐Tuberculous Mycobacteria and the Rise of *Mycobacterium abscessus* .” Nature Reviews Microbiology 18, no. 7: 392–407. 10.1038/s41579-020-0331-1. 32086501

[emi70465-bib-0053] Keesing, F. , R. D. Holt , and R. S. Ostfeld . 2006. “Effects of Species Diversity on Disease Risk.” Ecology Letters 9, no. 4: 485–498. 10.1111/j.1461-0248.2006.00885.x. 16623733

[emi70465-bib-0054] Keesing, F. , and R. S. Ostfeld . 2021. “Dilution Effect in Disease Ecology.” Ecology Letters 24, no. 11: 2490–2505. 10.1111/ele.13875. PMC9291114 34482609

[emi70465-bib-0115] Kirschner, R. A., Jr. , B. C. Parker , and J. O. Falkinham III . 1992. “Epidemiology of Infection by Nontuberculous Mycobacteria: Mycobacterium Avium, Mycobacterium Intracellulare, and Mycobacterium Scrofulaceum in Acid, Brown‐Water Swamps of the Southeastern United States and Their Association With Environmental Variables.” American Review of Respiratory Disease 145, no. 2_Part_1: 271–275. 10.1164/ajrccm/145.2_Pt_1.271. 1736730

[emi70465-bib-0055] Kirschner, R. A. , B. C. Parker , and J. O. Falkinham . 1999. “Humic and Fulvic Acids Stimulate the Growth of *Mycobacterium avium* .” FEMS Microbiology Ecology 30: 327–332. 10.1111/j.1574-6941.1999.tb00660.x. 10568841

[emi70465-bib-0056] Kox, L. F. , J. Van Leeuwen , S. Knijper , H. M. Jansen , and A. H. Kolk . 1995. “PCR Assay Based on DNA Coding for 16S rRNA for Detection and Identification of Mycobacteria in Clinical Samples.” Journal of Clinical Microbiology 33, no. 12: 3225–3233. 10.1128/jcm.33.12.3225-3233.1995. PMC228678 8586707

[emi70465-bib-0057] Lai, H. C. , T. H. Ng , M. Ando , et al. 2015. “Pathogenesis of Acute Hepatopancreatic Necrosis Disease (AHPND) in Shrimp.” Fish & Shellfish Immunology 47, no. 2: 1006–1014. 10.1016/j.fsi.2015.11.008. 26549178

[emi70465-bib-0058] Lamrabet, O. , and M. Drancourt . 2013. “ *Mycobacterium gilvum* Illustrates Size‐Correlated Relationships Between Mycobacteria and *Acanthamoeba polyphaga* .” Applied and Environmental Microbiology 79, no. 5: 1606–1611. 10.1128/AEM.03765-12. PMC3591941 23275502

[emi70465-bib-0059] Lauber, C. L. , M. Hamady , R. Knight , and N. Fierer . 2009. “Pyrosequencing‐Based Assessment of Soil pH as a Predictor of Soil Bacterial Community Structure at the Continental Scale.” Applied and Environmental Microbiology 75: 5111. 10.1128/AEM.00335-09. PMC2725504 19502440

[emi70465-bib-0060] Lavania, M. , R. Turankar , I. Singh , A. Nigam , and U. Sengupta . 2014. “Detection of *Mycobacterium gilvum* First Time From the Bathing Water of Leprosy Patient From Purulia, West Bengal.” International Journal of Mycobacteriology 3, no. 4: 286–289. 10.1016/j.ijmyco.2014.10.005. 26786629

[emi70465-bib-0061] Le Dantec, C. , J. P. Duguet , A. Montiel , N. Dumoutier , S. Dubrou , and V. Vincent . 2002. “Chlorine Disinfection of Atypical Mycobacteria Isolated From a Water Distribution System.” Applied and Environmental Microbiology 68, no. 3: 1025–1032. 10.1128/AEM.68.3.1025-1032.2002. PMC123737 11872446

[emi70465-bib-0116] Li, M. , L. Chen , F. Zhao , et al. 2023. “Effects of Urban–Rural Environmental Gradient on Soil Microbial Community in Rapidly Urbanizing Area.” Ecosystem Health and Sustainability 9: 0118. 10.34133/ehs.0118.

[emi70465-bib-0062] Liang, Y. , X. Zhang , J. Zhou , and G. Li . 2015. “Long‐Term Oil Contamination Increases Deterministic Assembly Processes in Soil Microbes.” Ecological Applications 25: e1243. 10.1890/14-1672.1. 26485952

[emi70465-bib-0063] Lin, L. , R. Du , Q. Wu , and Y. Xu . 2023. “Metabolic Cooperation Between Conspecific Genotypic Groups Contributes to Bacterial Fitness.” ISME Communications 3: 1–11. 10.1038/s43705-023-00250-8. PMC10147913 37117489

[emi70465-bib-0065] Lindeboom, J. A. , L. E. Bruijnesteijn van Coppenraet , D. van Soolingen , J. M. Prins , and E. J. Kuijper . 2011. “Clinical Manifestations, Diagnosis, and Treatment of *Mycobacterium haemophilum* Infections.” Clinical Microbiology Reviews 24, no. 4: 701–717. 10.1128/cmr.00020-11. PMC3194825 21976605

[emi70465-bib-0066] Mendes de Lima, C. A. , H. Magdinier Gomes , M. A. Cardoso Oelemann , et al. 2013. “Nontuberculous Mycobacteria in Respiratory Samples From Patients With Pulmonary Tuberculosis in the State of Rondônia, Brazil.” Memórias Do Instituto Oswaldo Cruz 108, no. 4: 457–462. 10.1590/0074-0276108042013010. PMC3970618 23827995

[emi70465-bib-0067] Morris, A. , R. Gozlan , E. Marion , et al. 2014. “First Detection of *mycobacterium ulcerans* DNA in Environmental Samples From South America.” PLoS Neglected Tropical Diseases 8, no. 1: e2660. 10.1371/journal.pntd.0002660. PMC3907311 24498449

[emi70465-bib-0068] Morris, A. L. , J.‐F. Guégan , D. Andreou , et al. 2016. “Deforestation‐Driven Food‐Web Collapse Linked to Emerging Tropical Infectious Disease, *Mycobacterium ulcerans* .” Science Advances 2, no. 12: e1600387. 10.1126/sciadv.1600387. PMC5142798 27957534

[emi70465-bib-0069] Mve‐Obiang, A. , R. E. Lee , F. Portaels , and P. L. Small . 2003. “Heterogeneity of Mycolactones Produced by Clinical Isolates of *Mycobacterium ulcerans* : Implications for Virulence.” Infection and Immunity 71, no. 2: 774–783. 10.1128/IAI.71.2.774-783.2003. PMC145382 12540557

[emi70465-bib-0070] Nadell, C. D. , K. Drescher , and K. R. Foster . 2016. “Spatial Structure, Cooperation and Competition in Biofilms.” Nature Reviews Microbiology 14: 589–600. 10.1038/nrmicro.2016.84. 27452230

[emi70465-bib-0071] Nemergut, D. R. , S. K. Schmidt , T. Fukami , et al. 2013. “Patterns and Processes of Microbial Community Assembly.” Microbiology and Molecular Biology Reviews 77, no. 3: 342–356. 10.1128/mmbr.00051-12. PMC3811611 24006468

[emi70465-bib-0072] Nichols, G. , T. Ford , J. Bartram , A. Dufour , and F. Portaels . 2004. “Pathogenic Mycobacteria in Water. A Guide to Public Health Consequences, Monitoring and Management.” In Emerging Issues in Water and Infectious Disease Series, edited by S. Pedley , J. Bartram , G. Rees , et al. World Health Organization by IWA Publishing.

[emi70465-bib-0073] Norton, C. D. , and M. W. LeChevallier . 2000. “A Pilot Study of Bacteriological Population Changes Through Potable Water Treatment and Distribution.” Applied and Environmental Microbiology 66: 268–276. 10.1128/AEM.66.1.268-276.2000. PMC91817 10618235

[emi70465-bib-0074] Nuñez, N. F. , L. Maggia , P.‐L. Stenger , et al. 2021. “Potential of High‐Throughput eDNA Sequencing of Soil Fungi and Bacteria for Monitoring Ecological Restoration in Ultramafic Substrates: The Case Study of the New Caledonian Biodiversity Hotspot.” Ecological Engineering 173: 106416. 10.1016/j.ecoleng.2021.106416.

[emi70465-bib-0075] Olds, B. P. , C. L. Jerde , M. A. Renshaw , et al. 2016. “Estimating Species Richness Using Environmental DNA.” Ecology and Evolution 6, no. 12: 4214–4226. 10.1002/ece3.2186. PMC4972244 27516876

[emi70465-bib-0076] Oloya, J. , J. Opuda‐Asibo , R. Kazwala , et al. 2008. “Mycobacteria Causing Human Cervical Lymphadenitis in Pastoral Communities in the Karamoja Region of Uganda.” Epidemiology and Infection 136, no. 5: 636–643. 10.1017/S095026880700900. PMC2870852 17599779

[emi70465-bib-0077] Ortiz‐Zayas, J. R. , E. Cuevas , O. L. Mayol‐Bracero , et al. 2006. “Urban Influences on the Nitrogen Cycle in Puerto Rico.” Biogeochemistry 79, no. 1: 109–133. 10.1007/s10533-006-9005-y.

[emi70465-bib-0078] Phung, T. N. , D. Caruso , S. Godreuil , N. Keck , T. Vallaeys , and J.‐C. Avarre . 2013. “Rapid Detection and Identification of Nontuberculous Mycobacterial Pathogens in Fish by Using High‐Resolution Melting Analysis.” Applied and Environmental Microbiology 79, no. 24: 7837–7845. 10.1128/AEM.00822-13. PMC3837818 24123734

[emi70465-bib-0079] Pitlik, S. D. , and O. Koren . 2017. “How Holobionts Get Sick–Toward a Unifying Scheme of Disease.” Microbiome 5: 64. 10.1186/s40168-017-0281-7. PMC5482958 28646902

[emi70465-bib-0080] Pont, D. , M. Rocle , A. Valentini , et al. 2018. “Environmental DNA Reveals Quantitative Patterns of Fish Biodiversity in Large Rivers Despite Its Downstream Transportation.” Scientific Reports 8, no. 1: 10361. 10.1038/s41598-018-28424-8. PMC6039509 29991759

[emi70465-bib-0081] Pontiroli, A. , T. T. Khera , B. B. Oakley , et al. 2013. “Prospecting Environmental Mycobacteria: Combined Molecular Approaches Reveal Unprecedented Diversity.” PLoS One 8, no. 7: e68648. 10.1371/journal.pone.0068648. PMC3715504 23874704

[emi70465-bib-0082] Poudel, R. , A. Jumpponen , D. Schlatter , et al. 2016. “Microbiome Networks: A Systems Framework for Identifying Candidate Microbial Assemblages for Disease Management.” Phytopathology 106: 1083–1096. 10.1094/PHYTO-02-16-0058-FI. 27482625

[emi70465-bib-0083] Primm, T. P. , C. A. Lucero , and J. O. Falkinham . 2004. “Health Impacts of Environmental Mycobacteria.” Clinical Microbiology Reviews 17, no. 1: 98–106. 10.1128/CMR.17.1.98-106.2004. PMC321467 14726457

[emi70465-bib-0084] Radomski, N. , A. Roguet , F. S. Lucas , et al. 2013. “ atpE Gene as a New Useful Specific Molecular Target to Quantify Mycobacterium in Environmental Samples.” BMC Microbiology 13: 1–13. 10.1186/1471-2180-13-277. PMC4219376 24299240

[emi70465-bib-0085] Reed, C. , C. F. Von Reyn , S. Chamblee , et al. 2006. “Environmental Risk Factors for Infection With *Mycobacterium avium* Complex.” American Journal of Epidemiology 164, no. 1: 32–40. 10.1093/aje/kwj159. 16675537

[emi70465-bib-0086] Rein, A. , I. K. Adam , A. Miltner , K. Brumme , M. Kästner , and S. Trapp . 2016. “Impact of Bacterial Activity on Turnover of Insoluble Hydrophobic Substrates (Phenanthrene and Pyrene)—Model Simulations for Prediction of Bioremediation Success.” Journal of Hazardous Materials 306: 105–114. 10.1016/j.jhazmat.2015.12.005. 26705887

[emi70465-bib-0087] Rejas, D. , K. Muylaert , and L. De Meester . 2005. “Nutrient Limitation of Bacteria and Sources of Nutrients Supporting Nutrient‐Limited Bacterial Growth in an Amazonian Floodplain Lake.” Aquatic Microbial Ecology 39, no. 1: 57–67. 10.3354/ame.

[emi70465-bib-0088] Reverter, M. , S. Sarter , D. Caruso , et al. 2020. “Aquaculture at the Crossroads of Global Warming and Antimicrobial Resistance.” Nature Communications 11: 1870. 10.1038/s41467-020-15735-6. PMC7170852 32312964

[emi70465-bib-0089] Riaz, T. , W. Shehzad , A. Viari , F. Pompanon , P. Taberlet , and E. Coissac . 2011. “ecoPrimers: Inference of New DNA Barcode Markers From Whole Genome Sequence Analysis.” 39, no. 21: e145. 10.1093/nar/gkr732. PMC3241669 21930509

[emi70465-bib-0090] Rissanen, A. J. , S. Peura , P. A. Mpamah , et al. 2019. “Vertical Stratification of Bacteria and Archaea in Sediments of a Small Boreal Humic Lake.” FEMS Microbiology Letters 366, no. 5: fnz044. 10.1093/femsle/fnz044. PMC6476745 30806656

[emi70465-bib-0091] Schnell, I. B. , K. Bohmann , and M. T. P. Gilbert . 2015. “Tag Jumps Illuminated—Reducing Sequence‐to‐Sample Misidentifications in Metabarcoding Studies.” Molecular Ecology Resources 15: 1289–1303. 10.1111/1755-0998.12402. 25740652

[emi70465-bib-0092] Selman, M. , A. Pardo , and T. E. King Jr. 2012. “Hypersensitivity Pneumonitis: Insights in Diagnosis and Pathobiology.” American Journal of Respiratory and Critical Care Medicine 186, no. 4: 314–324. 10.1164/rccm.201203-0513CI. 22679012

[emi70465-bib-0093] Shi, Y. , M. Delgado‐Baquerizo , Y. Li , et al. 2020. “Abundance of Kinless Hubs Within Soil Microbial Networks Are Associated With High Functional Potential in Agricultural Ecosystems.” Environment International 142: 105869. 10.1016/j.envint.2020.105869. 32593837

[emi70465-bib-0094] Stackebrandt, E. , and B. M. Goebel . 1994. “Taxonomic Note: A Place for DNA‐DNA Reassociation and 16S rRNA Sequence Analysis in the Present Species Definition in Bacteriology.” International Journal of Systematic and Evolutionary Microbiology 44: 846–849. 10.1099/00207713-44-4-846.

[emi70465-bib-0095] Stanford, J. L. , and W. J. Gunthorpe . 1971. “A Study of Some Fast‐Growing Scotochromogenic Mycobacteria Including Species Descriptions of *Mycobacterium gilvum* (New Species) and *Mycobacterium duvalii* (New Species).” British Journal of Experimental Pathology 52: 627–637. PMC2072389 5002706

[emi70465-bib-0096] Stat, M. , M. J. Huggett , R. Bernasconi , et al. 2017. “Ecosystem Biomonitoring With eDNA: Metabarcoding Across the Tree of Life in a Tropical Marine Environment.” Scientific Reports 7, no. 1: 12240. 10.1038/s41598-017-12501-5. PMC5612959 28947818

[emi70465-bib-0097] Straus, W. L. , S. M. Ostroff , D. B. Jemigan , et al. 1994. “Clinical and Epidemiologic Characteristics of *Mycobacterium haemophilum* , an Emerging Pathogen in Immunocompromised Patients.” Annals of Internal Medicine 120: 118–125. 10.7326/0003-4819-120-2-199401150-00004. 8256970

[emi70465-bib-0098] Tabah, E. N. , C. R. Johnson , H. Degnonvi , G. Pluschke , and K. Röltgen . 2019. “Buruli Ulcer in Africa.” In Buruli Ulcer: *Mycobacterium ulcerans* Disease, 43–60. Springer International Publishing. 10.1007/978-3-030-11114-4_2.

[emi70465-bib-0099] Taylor, R. H. , J. O. Falkinham , C. D. Norton , and M. W. LeChevallier . 2000. “Chlorine, Chloramine, Chlorine Dioxide, and Ozone Susceptibility of *Mycobacterium avium* .” Applied and Environmental Microbiology 66: 1702–1705. 10.1128/AEM.66.4.1702-1705.2000. PMC92045 10742264

[emi70465-bib-0100] Tobler, N. E. , M. Pfunder , K. Herzog , J. E. Frey , and M. Altwegg . 2006. “Rapid Detection and Species Identification of *Mycobacterium* spp. Using Real‐Time PCR and DNA‐Microarray.” Journal of Microbiological Methods 66, no. 1: 116–124. 10.1016/j.mimet.2005.10.016. 16360893

[emi70465-bib-0101] Toju, H. , K. G. Peay , M. Yamamichi , et al. 2018. “Core Microbiomes for Sustainable Agroecosystems.” Nature Plants 4: 247–257. 10.1038/s41477-018-0139-4. 29725101

[emi70465-bib-0102] Toju, H. , A. S. Tanabe , and H. Sato . 2018. “Network Hubs in Root‐Associated Fungal Meta‐Communities.” Microbiome 6: 116. 10.1186/s40168-018-0497-1. PMC6015470 29935536

[emi70465-bib-0103] Toranzo, A. E. 2004. Report About Fish Bacterial Diseases, 49–89. Mediterranean Aquaculture Laboratories.

[emi70465-bib-0104] Tortoli, E. 2006. “The New Mycobacteria: An Update.” FEMS Immunology and Medical Microbiology 48: 159–178. 10.1111/j.1574-695X.2006.00123.x. 17064273

[emi70465-bib-0105] Valentini, A. , P. Taberlet , C. Miaud , et al. 2016. “Next‐Generation Monitoring of Aquatic Biodiversity Using Environmental DNA Metabarcoding.” Molecular Ecology 25, no. 4: 929–942. 10.1111/mec.13428. 26479867

[emi70465-bib-0106] van Ingen, J. , M. J. Boeree , P. N. R. Dekhuijzen , and D. van Soolingen . 2009. “Environmental Sources of Rapid Growing Nontuberculous Mycobacteria Causing Disease in Humans.” Clinical Microbiology and Infection 15, no. 10: 888–893. 10.1111/j.1469-0691.2009.03013.x. 19845700

[emi70465-bib-0107] Wagg, C. , K. Schlaeppi , S. Banerjee , E. E. Kuramae , and M. G. A. van der Heijden . 2019. “Fungal‐Bacterial Diversity and Microbiome Complexity Predict Ecosystem Functioning.” Nature Communications 10: 4841. 10.1038/s41467-019-12798-y. PMC6813331 31649246

[emi70465-bib-0108] Wang, K. , X. Ye , H. Chen , et al. 2015. “Bacterial Biogeography in the Coastal Waters of Northern Zhejiang, East China Sea Is Highly Controlled by Spatially Structured Environmental Gradients.” Environmental Microbiology 17: 3898. 10.1111/1462-2920.12884. 25912020

[emi70465-bib-0109] Wit, R. , and T. Bouvier . 2006. “Everything Is Everywhere, but, the Environment Selects; What Did Baas Becking and Beijerinck Really Say?” Environmental Microbiology 8, no. 4: 755–758. 10.1111/j.1462-2920.2006.01017.x. 16584487

[emi70465-bib-0110] Wu, X. , Y. Lu , S. Zhou , L. Chen , and B. Xu . 2016. “Impact of Climate Change on Human Infectious Diseases: Empirical Evidence and Human Adaptation.” Environment International 86: 14–23. 10.1016/j.envint.2015.09.007. 26479830

[emi70465-bib-0111] Xiong, J. , Y. Liu , X. Lin , et al. 2012. “Geographic Distance and pH Drive Bacterial Distribution in Alkaline Lake Sediments Across Tibetan Plateau.” Environmental Microbiology 14: 2457. 10.1111/j.1462-2920.2012.02799.x. PMC3477592 22676420

[emi70465-bib-0112] Xiong, J. , X. Ye , K. Wang , et al. 2014. “Biogeography of the Sediment Bacterial Community Responds to a Nitrogen Pollution Gradient in the East China Sea.” Applied and Environmental Microbiology 80: 1919. 10.1128/AEM.03731-13. PMC3957648 24413606

[emi70465-bib-0113] Yanong, R. P. E. , D. B. Pouder , and J. O. Falkinham . 2010. “Association of Mycobacteria in Recirculating Aquaculture Systems and Mycobacterial Disease in Fish.” Journal of Aquatic Animal Health 22, no. 4: 219–223. 10.1577/H10-009.1. 21413504

